# Sterically Controlled Interfacial Charge‐Transfer Mechanisms in Unsymmetrical Squaraine Dyes for Suppressed Aggregation and Enhanced Performance in High‐Efficiency Dye‐Sensitized Solar Cells

**DOI:** 10.1002/open.70199

**Published:** 2026-04-20

**Authors:** Abdullah N. Alotaibi, Sultan A. Al‐horaibi, Yaaser Q. Almulaiky, Ali Arabie

**Affiliations:** ^1^ Department of Chemistry Imam Mohammad Ibn Saud Islamic University (IMSIU) Riyadh Saudi Arabia; ^2^ Department of Chemistry Albaydha University Albaydha City Yemen; ^3^ Department of Biochemistry The Applied College University of Jeddah Jeddah Saudi Arabia

**Keywords:** Π‐extended squaraines, alkyl chain engineering, charge recombination, coadsorbent cDCA, density functional theory

## Abstract

Steric regulation at the dye/semiconductor interface critically governs charge recombination and interfacial energetics in dye‐sensitized solar cells (DSSCs) but remains poorly defined for unsymmetrical squaraine sensitizers. Here, two π‐extended unsymmetrical squaraine dyes (AQ1 and AQ2) are designed to clarify how alkyl‐chain‐induced steric modulation influences aggregation, surface packing, and intramolecular charge‐transfer (ICT) dynamics. Both dyes show strong visible–near‐infrared absorption and suitable the highest occupied molecular orbital and the lowest unoccupied molecular orbital (HOMO–LUMO) alignment for electron injection into TiO_2_. In AQ2, branched alkyl substituents provide enhanced steric shielding at the TiO_2_ interface, suppressing aggregation, modifying interfacial dipoles, and increasing *R*_rec_. Importantly, efficiency gains arise mainly from steric control of interfacial energetics and recombination kinetics rather than aggregation suppression alone. Consequently, AQ2‐based DSSCs deliver 7.41% power conversion efficiency with higher *R*_rec_ = 11.64 Ω and longer electron lifetime (*τ* = 8.58 ms) than AQ1, despite lower dye loading. Electrochemical impedance spectroscopy (EIS) and density functional theory (DFT) corroborate suppressed back‐electron transfer.

## Graphical abstract

Molecular engineering of cis‐configured unsymmetrical squaraine dyes reveals how branched alkyl substituents regulate aggregation and interfacial charge–transfer processes on TiO_2_. Steric shielding suppresses H‐type aggregation and retards interfacial recombination, leading to enhanced recombination resistance and improved open‐circuit voltage. These results establish steric modulation as a key design parameter for high‐performance dye‐sensitized solar cell sensitizers.

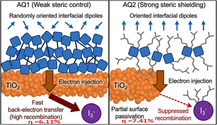

## 1 Introduction

Dye‐sensitized solar cells (DSSCs) continue to attract considerable attention as a versatile photovoltaic technology owing to their low‐cost fabrication, mechanical flexibility, and molecularly tunable photoactive components. Despite the remarkable progress achieved with symmetrical squaraine dyes, the role of steric engineering in unsymmetrical squaraine sensitizers for controlling interfacial aggregation and recombination remains insufficiently understood. Recent studies have demonstrated the importance of molecular packing and dipole orientation in DSSCs. However, systematic investigations of sterically regulated intramolecular charge–transfer (ICT) processes in unsymmetrical squaraine dyes are still limited. Therefore, this article aims to clarify how alkyl‐chain steric modulation influences aggregation behavior and charge–transfer dynamics at the TiO_2_ interface [1, 2, 3]. In particular, charge recombination between injected electrons in TiO_2_ and oxidized dye molecules or redox species in the electrolyte remains a dominant efficiency‐limiting pathway, directly impacting the open‐circuit voltage (*V*_oc_) and consequently power conversion efficiency (*η*) [4, 5, 6]. Molecular engineering of organic sensitizers has emerged as an effective strategy to regulate ICT processes. Approaches such as π‐conjugation extension, optimization of donor–acceptor frameworks, and coadsorbent incorporation have been widely employed to enhance light absorption and suppress aggregation‐induced quenching [7, 8, 9, 10]. Among these strategies, steric modification through alkyl‐chain engineering has proven particularly effective in influencing dye orientation, surface coverage, and recombination dynamics by modulating the spatial arrangement of dyes on the TiO_2_ surface [11, 12, 13, 14]. However, in many reported systems, steric effects are treated empirically as a means to reduce aggregation, rather than being analyzed as a fundamental parameter governing interfacial energetics [15, 16, 17, 18]. Although numerous squaraine sensitizers have been reported for DSSCs, most studies focus on symmetrical architectures or introduce bulky substituents as secondary aggregation suppressors without isolating their mechanistic role [19]. In contrast, truly π‐extended unsymmetrical squaraine dyes incorporating sterically differentiated donor‐side alkyl architectures remain scarcely explored, particularly in the context of interfacial recombination kinetics. Importantly, no prior study has systematically designed unsymmetrical squaraines in which steric shielding is deliberately varied while preserving an identical π‐conjugated backbone to decouple steric effects from intrinsic electronic contributions. Squaraine dyes represent a compelling class of organic sensitizers due to their intense visible–near‐infrared absorption, high molar extinction coefficients, and compact donor–acceptor electronic structures [20, 21, 22, 23]. These characteristics make squaraines especially attractive for enhancing photocurrent generation in DSSCs [24]. Nevertheless, their highly planar π‐conjugated backbones often promote strong intermolecular π–π stacking, leading to pronounced aggregation on semiconductor surfaces and accelerated charge recombination [1, 25]. Various molecular strategies—including bulky donor groups, branched alkyl substituents, and coadsorbents such as chenodeoxycholic acid (CDCA)—have been applied to mitigate these effects, yielding moderate improvements in device performance [26, 27]. Despite these advances, a critical mechanistic gap persists in the design of unsymmetrical squaraine sensitizers. Previous studies generally correlate steric modification with improved photovoltaic metrics, yet do not establish a causal link between steric architecture, interfacial dipole formation, recombination resistance (*R*_rec_), and tau [28, 29]. In particular, steric effects are often inseparable from changes in conjugation length, anchoring mode, or electronic structure. Consequently, the fundamental question of whether steric shielding alone can regulate interfacial energetics—independent of dye loading and intrinsic frontier orbital alignment—remains unresolved [30, 31, 32]. In this work, we present two π‐extended unsymmetrical squaraine dyes, AQ1 and AQ2, specifically designed to elucidate the mechanistic role of steric alkyl‐chain engineering at the TiO_2_ interface. Both dyes share an identical conjugated backbone comprising an indoline donor, a thiophene π‐spacer, and a cyanoacrylic acid anchoring group to ensure comparable electronic structures and light‐harvesting characteristics. The key structural distinction lies in the donor‐side alkyl substituents: AQ1 contains relatively short alkyl chains, whereas AQ2 incorporates branched long‐chain alkyl groups that introduce pronounced steric shielding. By combining spectroscopic characterization, electrochemical analysis, density functional theory (DFT), and electrochemical impedance spectroscopy (EIS), we demonstrate that steric modulation fundamentally alters ICT behavior. The branched alkyl chains in AQ2 regulate dye packing, modify interfacial dipole interactions, suppress charge recombination, and prolong *τ*—leading to enhanced *V*_oc_ and *η*. Rather than presenting steric modification as a secondary structural detail, this article establishes steric control as a primary interfacial design parameter for regulating recombination kinetics in squaraine‐based DSSCs. The insights derived herein provide practical molecular design guidelines for developing next‐generation organic sensitizers tailored for efficient and stable solar energy conversion.

## 2 Results and Discussion

### 2.1 Molecular Design Rationale and Structural Considerations

**SCHEME 1:**
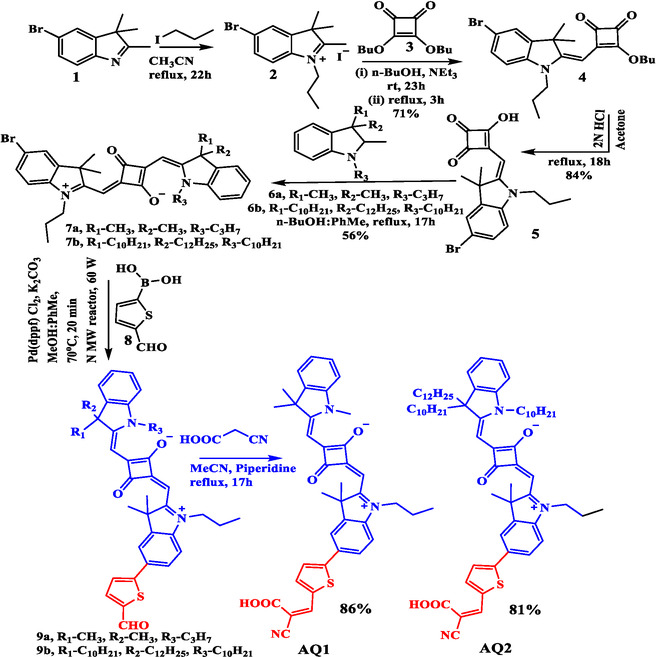
Synthetic routes and molecular structures of AQ1 and AQ2.

The molecular structures of AQ1 and AQ2 were deliberately designed to isolate the influence of steric modulation on ICT behavior while preserving comparable electronic structures. Both dyes share an identical π‐extended conjugated framework comprising an indoline donor, a thiophene π‐spacer, and a cyanoacrylic acid anchoring group, ensuring similar light‐harvesting characteristics and frontier orbital alignment. The only structural difference lies in the donor‐side alkyl substituents: AQ1 bears relatively short alkyl chains, whereas AQ2 incorporates branched long‐chain alkyl groups that provide enhanced steric shielding at the TiO_2_/dye/electrolyte interface. Because dye loading, anchoring functionality, and π‐conjugation length are otherwise comparable, this design enables a direct evaluation of steric effects on dye packing, interfacial dipole formation, and charge recombination kinetics without significant electronic perturbation. Notably, AQ1 and AQ2 form a deliberately constructed steric‐control pair rather than routine alkyl variants, representing—to the best of our knowledge—the first controlled steric differentiation strategy applied to π‐extended unsymmetrical squaraine sensitizers in DSSCs. The synthetic routes and chemical structures of AQ1 and AQ2 are illustrated in Scheme 1.

### 2.2 Optical Properties and Aggregation Behavior

**FIGURE 1:**
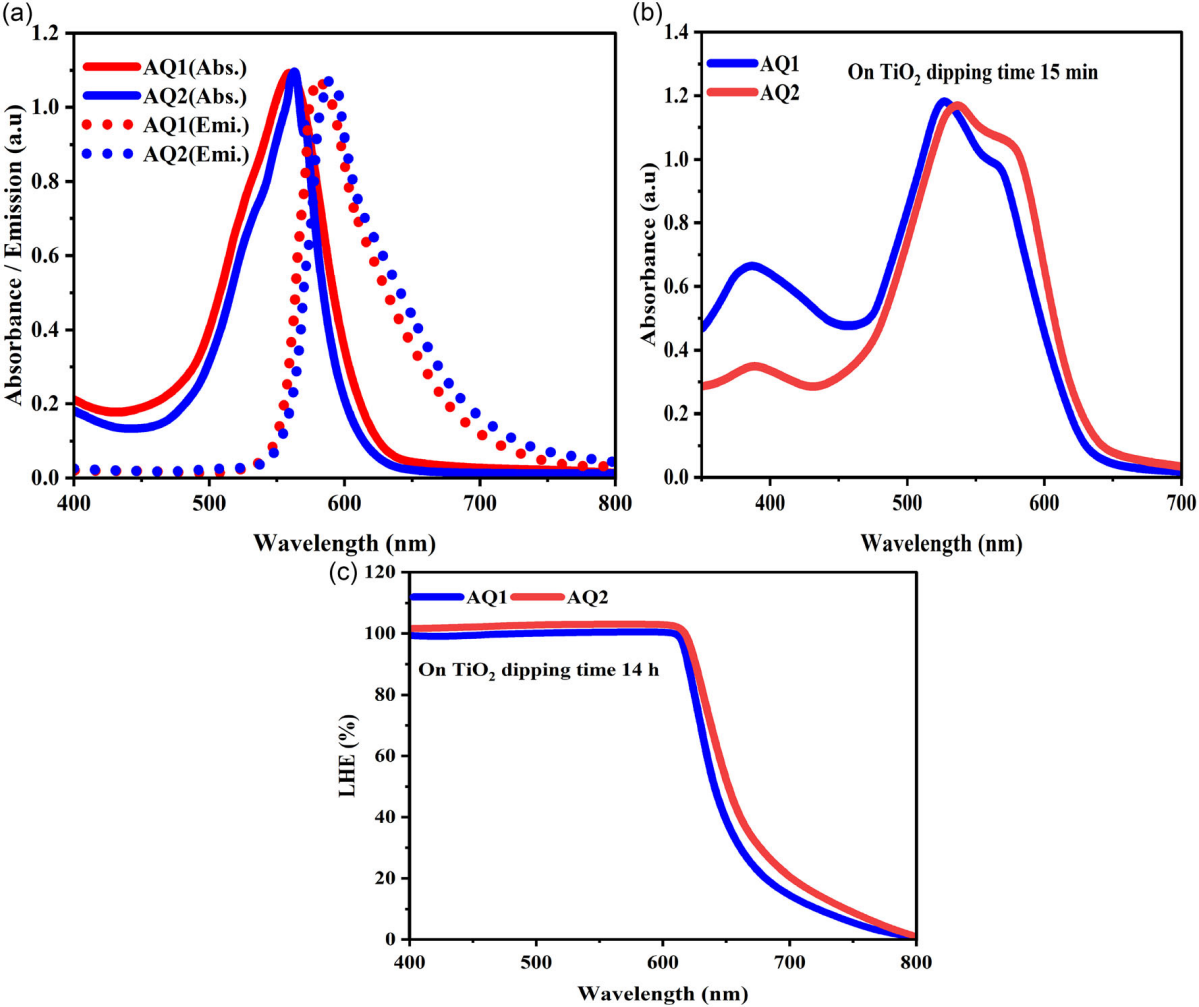
(a) UV–Vis absorption and fluorescence spectra of AQ1 and AQ2 in acetonitrile (CH_3_CN) soultion. (b) Absorption spectra of TiO_2_ films sensitized with AQ1 and AQ2 after 15 min immersion. (c) Light‐harvesting efficiency (LHE) spectra of AQ1‐ and AQ2‐sensitized TiO_2_ films recorded after 14 h dipping time.

The absorption spectra of AQ1 and AQ2 were recorded in CH_3_CN solution to evaluate their intrinsic photophysical properties. Both dyes exhibit intense absorption in the visible–near‐infrared region originating from ICT transitions along their extended π‐conjugated squaraine backbones. In solution, AQ1 and AQ2 display absorption maxima at 559 and 562 nm, respectively, accompanied by high molar extinction coefficients exceeding 1.1 × 10^5^ M^−1^ cm^−1^, highlighting their strong light‐harvesting capability. The corresponding fluorescence emission maxima appear at 583 nm for AQ1 and 589 nm for AQ2, indicating moderate Stokes shifts consistent with efficient ICT excited states (Figure 1a, Table 1). These results confirm that both chromophores possess excellent optical properties suitable for application as photosensitizers in DSSCs.

**TABLE 1:** Summary of key optoelectronic parameters of AQ1 and AQ2 sensitizers measured at RT.

Dyes	a *λ*_max_,nm/DFT	b *λ*_max_ (TiO_2_), nm	*ε*, M^−1^ cm^−1^, 10^5^	c *λ*_em,_ nm	LHE Δ*λ* (100%), nm	d *E*_oxd/onset_ (V vs. Ag/Ag^+^)	*E*_HOMO_ (V vs. NHE), V	*E*_LUMO_, V	e *E*_g_, eV	E_g_/DFT, eV
AQ1	559/541	525,565	1.17	583	164	0.422	0.952	−1.218	2.17	2.12
AQ2	562/549	531,577	1.19	589	175	0.406	0.936	−1.204	2.14	2.11

a–c Absorption and photoluminescence spectra were measured in CH_3_CN at RT.

d Oxidation potential (*E*_ox_) was determined by CV in CDM.

e *E*_g_ was estimated from the UV–Vis/emission intersection: *E*_g_ = 1240/*λ*_max_ (nm).

To further elucidate the aggregation behavior on semiconductor surfaces, the absorption spectra of AQ1‐ and AQ2‐sensitized TiO_2_ films were analyzed through Gaussian deconvolution. The spectra could be resolved into two principal components corresponding to monomeric and H‐aggregated dye species. The blueshifted band located at approximately 525–531 nm was attributed to H‐type aggregates arising from face‐to‐face π–π stacking interactions, whereas the longer‐wavelength band at approximately 565–577 nm corresponds to the monomeric ICT transition of the adsorbed dye molecules. Semiquantitative analysis of the peak areas indicates that AQ1 contains a larger aggregated fraction (∼55%), whereas AQ2 exhibits a higher proportion of monomeric species (∼65%). This behavior can be attributed to the presence of bulky branched alkyl substituents in AQ2, which provide effective steric shielding that inhibits close intermolecular π–π interactions and promotes a more isolated adsorption configuration on the TiO_2_ surface.

Consistent with this interpretation, AQ1 displays more pronounced spectral broadening and partial attenuation of the main absorption band after surface immobilization, indicating stronger intermolecular coupling and aggregation. Although some spectral variation may arise from heterogeneity in surface coverage, the systematic differences between the two dyes strongly suggest that aggregation effects dominate the observed spectral changes. The UV–Vis spectra of dye‐sensitized TiO_2_ films show absorption maxima at approximately 565 and 577 nm for AQ1 and AQ2, respectively, further confirming the presence of H‐type aggregation in both systems, with AQ1 exhibiting a more pronounced aggregation tendency. Additionally, the persistent absorption band observed at approximately 401–403 nm indicates that the thiophene‐centered π–π* transition remains intact after adsorption onto the semiconductor surface.

The calculated LHE spectra reveal broad photoresponse windows extending to approximately 164 and 173 nm for AQ1 and AQ2, respectively (Figure 1c). The slightly narrower spectral distribution observed for AQ2 suggests reduced intermolecular electronic coupling and suppressed aggregation at the TiO_2_ interface. Consequently, steric modulation in AQ2 effectively mitigates excessive aggregation while preserving strong optical absorption, thereby facilitating more efficient photon harvesting and interfacial charge generation in DSSCs. Nevertheless, although aggregation effects account for part of the optical differences between the two dyes, the superior photovoltaic performance of AQ2 cannot be attributed solely to aggregation suppression. A comprehensive understanding therefore requires examination of interfacial charge recombination dynamics.

**FIGURE 2:**
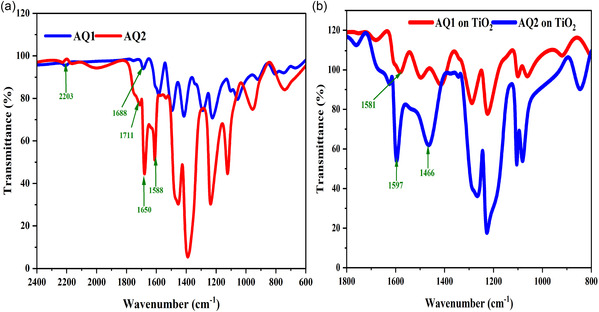
(a) FTIR spectra of AQ1 and AQ2 dyes and (b) FTIR spectra of AQ1 and AQ2 dyes on the TiO_2_ surface.

FTIR spectra of AQ1 and AQ2 were recorded to investigate their binding interactions with the TiO_2_ surface. The free dyes exhibited characteristic vibrations of the squaraine framework and anchoring groups. AQ1 showed a C=O stretching vibration of the carboxylic acid group at 1688 cm^−1^ together with squaric carbonyl bands at 1649 and 1586 cm^−1^, whereas AQ2 displayed analogous absorptions at 1711, 1650, and 1588 cm^−1^. Both dyes also exhibited a distinct C≡N stretching vibration near 2203 cm^−1^, confirming the presence of the cyanoacrylic acid anchoring unit (Figure 2a). Upon adsorption onto TiO_2_ films, noticeable spectral changes were observed (Figure 2b). The disappearance or significant shift of the carboxylic C=O band together with the appearance of bands at 1634 cm^−1^ (AQ1) and 1721 cm^−1^ (AQ2) indicates deprotonation of the carboxylic acid group and strong coordination of the resulting carboxylate with surface Ti^4+^ centers. The squaric carbonyl vibrations also shifted slightly to 1628 and 1595–1573 cm^−1^, reflecting modification of the electronic environment upon adsorption. Importantly, Additionally, the C–N stretching vibration shifts from ∼1345 cm^−1^ in the free dye to ∼1328 cm^−1^ after adsorption, suggesting secondary interaction between the amino group and the TiO_2_ surface. These spectral changes collectively indicate that the dye molecules are strongly anchored to the TiO_2_ surface through the carboxylate group, whereas the C–N donor moiety contributes to interfacial electronic coupling that facilitates efficient charge transfer in DSSCs.

### 2.3 Energy‐Level Alignment and Electrochemical Characteristics

**FIGURE 3:**
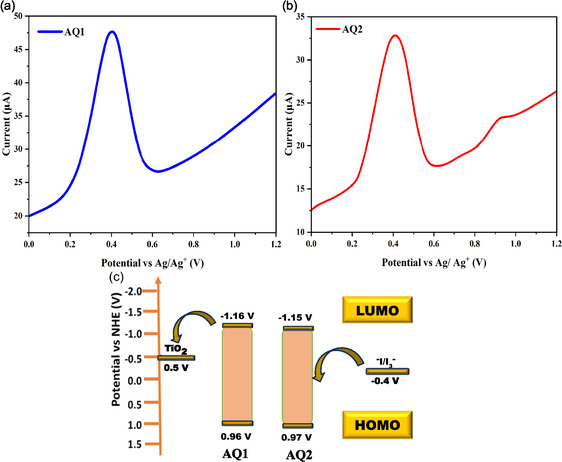
(a,b) Differential pulse voltammograms of AQ1 and AQ2 recorded in CH_2_Cl_2_. (c) Schematic HOMO–LUMO energy level alignment of AQ1 and AQ2 relative to the TiO_2_ conduction band and the I^‐^/I_3_^‐^ redox couple.

The electrochemical properties of AQ1 and AQ2 were investigated in anhydrous CH_2_Cl_2_ to determine their redox behavior and frontier orbital energy levels relevant to DSSCs operation. Differential pulse voltammetry (DPV), referenced to Fc/Fc^+^ and converted to the normal hydrogen electrode (NHE) scale (+0.70 V), reveals well‐defined oxidation processes for both dyes (Figure 3a–b), corresponding to highest occupied molecular orbital (HOMO) oxidation.

AQ1 and AQ2 exhibit similar HOMO energy levels at 0.952 and 0.936 V versus NHE, respectively (Table 1). Using optical bandgaps (*E*_g_) derived from absorption–emission overlap, the corresponding lowest unoccupied molecular orbital (LUMO) levels were estimated at −1.218 V (AQ1) and −1.204 V versus NHE (AQ2). The electrochemical *E*_g_ (2.17 eV for AQ1 and 2.14 eV for AQ2) closely matches the optical values, confirming the reliability of the measurements.

Both dyes exhibit favorable energetic alignment for DSSCs, with LUMO levels well above the TiO_2_ conduction band, providing sufficient driving force (∼0.5 V) for efficient electron injection, and HOMO levels suitably positioned below the I^‐^/I_3_^‐^ redox potential to ensure rapid dye regeneration (∼0.4 V). The slightly deeper HOMO of AQ2 suggests enhanced oxidative stability; however, the higher *V*_oc_ is primarily attributed to suppressed charge recombination, as evidenced by the increased *R*_rec_ observed in EIS measurements. Moreover, the close similarity in frontier orbital energies indicates that steric modification does not significantly affect the intrinsic redox properties of the dyes (Figure 3c). Therefore, differences in photovoltaic performance are primarily attributed to interfacial effects, particularly reduced aggregation and improved surface packing in AQ2, rather than variations in charge injection or regeneration kinetics.

### 2.4 Theoretical Investigation of Charge–Transfer Properties

**FIGURE 4:**
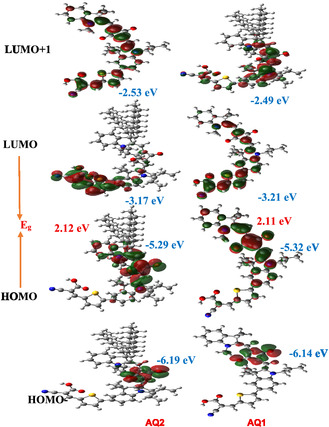
FMO distributions of AQ1 and AQ2 sensitizers computed via DFT at the B3LYP/6‐311G (d,p) Level.

DFT and TD‐DFT calculations were performed at the B3LYP/6‐311G(d, p) level using the IEFPCM (DCM) solvation model to investigate the electronic structures of AQ1 and AQ2. The calculated frontier molecular orbital distributions are very similar for both dyes (Figure 4). In both systems, the HOMO^–1^ is mainly localized on the indoline donor and the thiophene spacer, whereas the HOMO is delocalized over the donor unit and the squaraine core. The LUMO is predominantly distributed on the cyanoacrylic acid acceptor and the thiophene bridge, confirming efficient ICT from the donor toward the anchoring acceptor upon photoexcitation. The LUMO^+^^1^ extends over the squaraine core and indoline unit, reflecting the extended π‐conjugated framework of the dyes.

The calculated total dipole moments are 7.603 D for AQ1 and 8.312 D for AQ2, indicating slightly stronger charge separation in AQ2. Considering the most probable adsorption configuration through the cyanoacrylic acid anchoring group, the dipole moment component perpendicular to the TiO_2_ surface (μ⊥) was estimated to be approximately 4.21 D for AQ1 and 4.86 D for AQ2. The larger perpendicular dipole component in AQ2 can generate a stronger interfacial dipole at the dye/TiO_2_ interface, which may influence the position of the TiO_2_‐ CB edge and consequently affect the *V*_oc_.

The computed HOMO–LUMO energy gaps are very similar (2.12 eV for AQ1 and 2.11 eV for AQ2), consistent with their closely related molecular structures. TD‐DFT simulations predict dominant absorption transitions in the range of 541–549 nm, which are in good agreement with the experimentally observed absorption maxima at 559–562 nm. This agreement between theoretical and experimental optical transitions confirms the reliability of the computational model in describing the electronic structure and photophysical behavior of the dyes.

The electrochemical energy levels derived from cyclic voltammetry (HOMO/LUMO: 0.952/−1.218 V for AQ1 and 0.936/−1.204 V vs. NHE for AQ2) are consistent with the DFT‐calculated trends and indicate sufficient thermodynamic driving force for electron injection into the TiO_2_‐ CB. Notably, AQ2 exhibits slightly enhanced steric hindrance due to its branched alkyl substituents, which helps suppress intermolecular aggregation on the TiO_2_ surface. This effect contributes to improved oxidative stability, more uniform interfacial organization, and reduced charge recombination, ultimately leading to the observed enhancement in *V*_o__c_ compared with AQ1.

Importantly, the superior photovoltaic performance of AQ2 is achieved despite its lower dye‐loading density, demonstrating that the efficiency enhancement does not originate from increased surface coverage. Instead, each adsorbed AQ2 molecule contributes more effectively to charge separation and charge collection, indicating a steric‐efficiency‐per‐molecule advantage rather than a simple increase in light harvesting.

Although explicit TiO_2_–dye interface models were not considered in the present calculations, the computed dipole moments and frontier orbital distributions provide reliable insight into the intrinsic charge‐separation characteristics of the dyes. These theoretical results correlate well with the experimentally observed recombination behavior obtained from EIS, supporting the proposed structure–property–performance relationship. The calculations were therefore performed on isolated dye molecules to evaluate their intrinsic electronic and optical properties, whereas interfacial effects were primarily addressed through experimental spectroscopic and electrochemical measurements.

### 2.5 Photovoltaic Performance and Role of Steric Modulation

**FIGURE 5:**
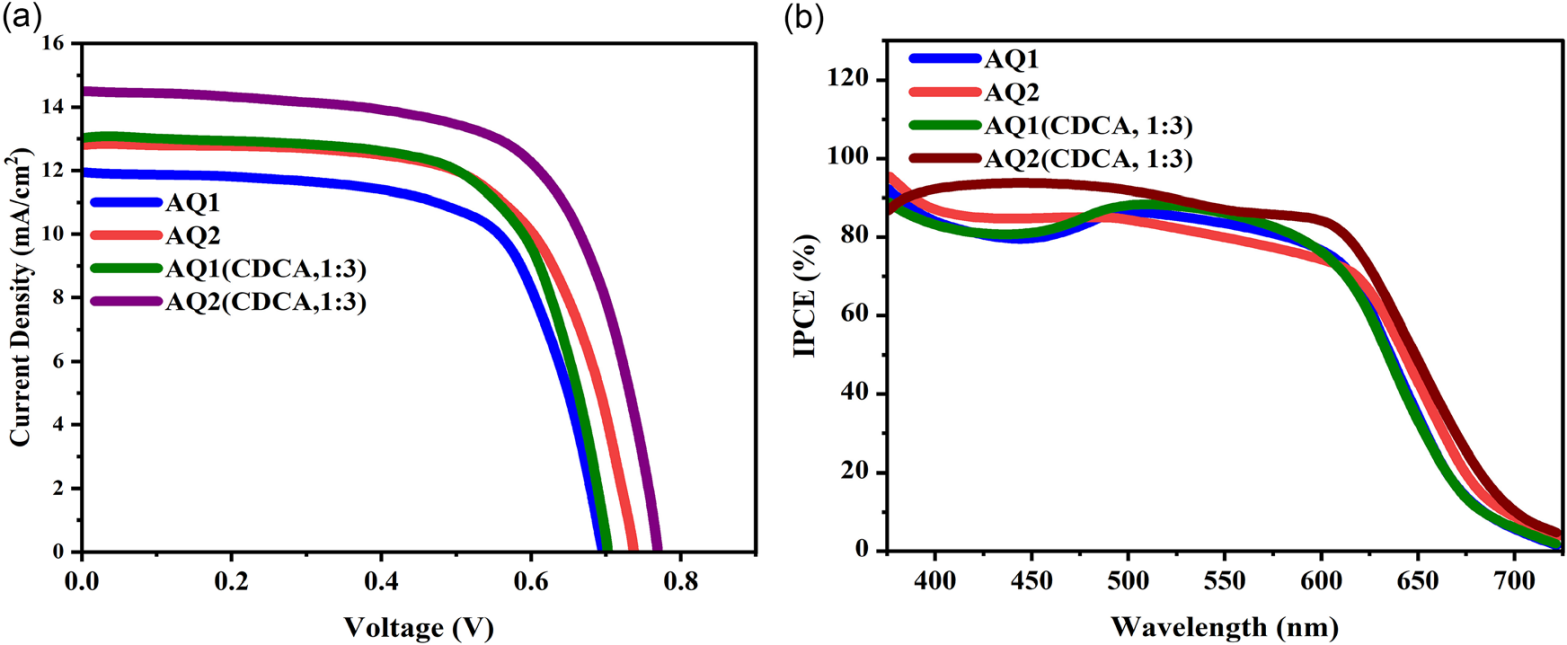
(a) *J*–*V* characteristics of DSSCs sensitized with AQ1 and AQ2, measured with and without 3 mM CDCA, illustrating the enhancement in *J*_sc_, *V*_oc_, and *η* upon aggregation suppression. (b) IPCE spectra of AQ1‐ and AQ2‐based DSSCs showing improved photocurrent response across the visible region after CDCA addition, confirming reduced H‐aggregation and enhanced electron injection efficiency. CDCA, chenodeoxycholic acid.

The photovoltaic performance of AQ1‐ and AQ2‐based DSSCs was evaluated with and without CDCA (Table 2, Figure 5a). Without CDCA, AQ2 already outperforms AQ1, delivering higher *J*_sc_ (13.05 vs. 11.95 mA cm^−2^) and *V*_oc_ (0.74 vs. 0.69 V), attributed to reduced aggregation induced by its sterically hindered structure. Incorporating 3 mM CDCA further enhances device performance for both dyes; however, AQ2 maintains its advantage, achieving a maximum efficiency of 7.41%, surpassing AQ1 (6.11%). In contrast, AQ2 exhibits lower dye‐loading density, confirming that improved performance arises from superior interfacial charge management rather than increased surface coverage. This indicates that each adsorbed AQ2 molecule contributes more effectively to charge separation and collection than AQ1, highlighting improved efficiency per dye molecule rather than higher surface density. The integrated photocurrent densities calculated from the incident photon‐to‐current efficiency (IPCE) spectra (Table 2) closely match the *J*_sc_ values obtained from the *J*–*V* measurements. For example, AQ2 exhibits an integrated current density of 14.55 mA cm^−2^ compared with a measured *J*_sc_ of 14.54 mA cm^−2^, showing excellent agreement and confirming the reliability of the photovoltaic measurements. Compared to the reference dye SQ‐220 (*η* = 4.02%) [33], both AQ1 and AQ2 demonstrate superior photovoltaic behavior, with AQ2 delivering nearly double the efficiency due to its optimized steric architecture and improved charge *R*_rec_. Although AQ2 exhibits a significantly higher *R*_rec_, the FF does not show a proportional increase because FF in DSSCs is influenced by multiple resistive components, including series resistance, charge transport resistance within the TiO_2_ network, and electrolyte diffusion. Consequently, the improved *R*_rec_ primarily enhances *V*_oc_ and suppresses recombination losses, whereas the overall FF remains governed by the combined internal resistances of the device. Consequently, AQ2 demonstrates the highest photovoltaic performance among the studied dyes, outperforming AQ1 and the reference dye SQ‐220 [33]. The enhanced efficiency of AQ2—reaching 7.41% with CDCA—stems from its sterically optimized structure, which effectively suppresses aggregation, improves *V*_oc_, and minimizes interfacial recombination. Although AQ2 exhibits lower dye‐loading density, its superior charge injection and reduced recombination per adsorbed molecule result in significantly improved device efficiency. These findings highlight the importance of steric modulation and coadsorbent engineering in achieving high‐performance DSSCs.

**TABLE 2:** The comparative study of photovoltaic parameters, dye loading, molecular footprint, and surface coverage. DSSCs fabricated using AQ1 and AQ2 sensitizers, compared to SQ‐220^38^.

Dyes	*CDCA*, mM	a *J*_sc_, mA/cm^2^	bIntegrated *J*_SC_, mA/cm^2^	*V*_oc_, V	FF	*η*, %	Dye loading Γ, mol cm^−2^	cMolecular footprint A, nm^2^ molecule^−1^	Surface coverage, molecules/nm^2^
AQ1	0	11.95	12.67	0.69	0.66	5.44	5.77 × 10^−7^	0.29	3.48
	3	12.84	12.98	0.71	0.67	6.11	5.12 × 10^−7^	0.32	3.13
AQ2	0	13.05	13.10	0.74	0.67	6.27	3.46 × 10^−7^	0.48	2.08
	3	14.54	14.55	0.77	0.68	7.41	2.96 × 10^−7^	0.56	1.82
SQ‐220		10.04		0.65	0.62	4.02	1.02 × 10^−7^		

a *J*_sc_ Values from *I*–*V* curves.

b Integrated photocurrent density calculated from the IPCE spectra. The device active area was 0.23 cm^2^, defined using a black mask during *J*–*V* measurements.

c The molecular footprint (A) was estimated using $A=1/(N_{A}\text{Γ})$, where $N_{A}$ is Avogadro's number and Γis the dye‐loading density obtained from desorption measurements.

Dye‐loading measurements were carried out by desorbing the sensitized TiO_2_ electrodes in 0.1 M NaOH solution in H_2_O/ethanol (1:1 v/v) for 30 min, followed by UV–Vis quantification of the released dyes. The dye‐loading densities (Γ) indicate that AQ1 exhibits higher adsorption than AQ2 under both additive‐free and CDCA‐containing conditions (Table 1). Without CDCA, AQ1 displays a dye loading of 5.77 × 10^−7^ mol cm^−2^, whereas AQ2 shows a lower value of 3.46 × 10^−7^ mol cm^−2^, reflecting reduced surface packing for the sterically bulkier dye.

The addition of 3 mM CDCA slightly decreases the dye‐loading densities to 5.12 × 10^−7^ mol cm^−2^ for AQ1 and 2.96 × 10^−7^ mol cm^−2^ for AQ2. This modest reduction is attributed to competitive adsorption between dye molecules and CDCA on the TiO_2_ surface, a well‐established strategy to mitigate dye aggregation. Importantly, both dyes retain efficient surface coverage while benefiting from improved interfacial organization.

To quantitatively assess the packing behavior of the adsorbed dyes, the molecular footprint (A) was calculated using the following equation:

$$A=\frac{1}{N_{A}\text{Γ}}$$

where $N_{A}$ is Avogadro's number and Γ is the dye surface concentration. Under additive‐free conditions, the molecular footprints are 0.29 nm^2^ molecule^−1^ for AQ1 and 0.48 nm^2^ molecule^−1^ for AQ2. In the presence of CDCA, the footprints increase slightly to 0.32 nm^2^ and 0.56 nm^2^ molecule^−1^, respectively. The increase reflects partial occupation of surface sites by the co‐adsorbent, which expands intermolecular spacing between adsorbed dye molecules.

Surface coverage, expressed in molecules nm^−2^, was also estimated for additional mechanistic insight:

$$\text{Surface coverage}=\frac{\text{Γ}\times N_{A}}{10^{14}}$$

AQ1 exhibits higher coverage (3.48 molecules/nm^2^ without CDCA, 3.13 molecules/nm^2^ with CDCA) compared to AQ2 (2.08 molecules/nm^2^ without CDCA, 1.82 molecules/nm^2^ with CDCA), consistent with the steric influence of the branched alkyl substituents in AQ2 that limit dense packing.

The larger molecular footprint and reduced surface coverage of AQ2 are advantageous: steric hindrance suppresses intermolecular π–π stacking and limits direct contact between the electrolyte and the semiconductor, thereby reducing charge recombination at the TiO_2_/electrolyte interface. This sterically regulated adsorption explains the observed improvements in *V*_oc_ and *J*_sc_. All calculated footprint values fall within the typical range for organic DSSC sensitizers (∼0.3–1.0 nm^2^ per molecule), confirming well‐defined monolayer formation.

Collectively, these results demonstrate that steric engineering in AQ2 not only modulates interfacial packing but also provides a rational design principle for squaraine dyes, where controlled intermolecular spacing directly enhances charge–transfer dynamics and overall DSSC performance.

### 2.6 ICT Kinetics: EIS Analysis

**FIGURE 6:**
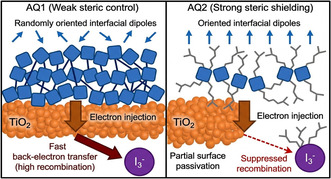
Steric‐controlled ICT mechanism in AQ1‐ and AQ2‐sensitized DSSCs.

EIS provides direct experimental evidence that steric alkyl‐chain engineering fundamentally governs ICT kinetics in unsymmetrical squaraine‐based DSSCs, rather than merely acting as an aggregation–suppression strategy (Figure 6). The measurements were performed at an applied bias of 0.5 V in the dark to enable direct comparison of recombination parameters under identical experimental conditions. Although comparison at a constant relative potential (*V*_applied_ *− V*_oc_) can provide additional insight, identical bias conditions were used for all devices to ensure that the observed trends reflect intrinsic differences in interfacial recombination behavior between the two sensitizers. The EIS parameters (*R*_rec_, *C* *_μ_*, and *τ*) are summarized in Table 3.

**TABLE 3:** Fitted EI parameters for DSSCs based on AQ1 and AQ2 sensitizers, measured at an applied potential of 0.5 V in the dark.

Dyes	*CDCA*, mM	*R*_rec_, Ω	*C*_µ_, mF	*T*, ms
AQ1	0	6.86	0.88	6.04
	3	10.28	0.58	5.96
AQ2	0	10.83	0.92	9.96
	3	11.64	0.73	8.58

**FIGURE 7:**
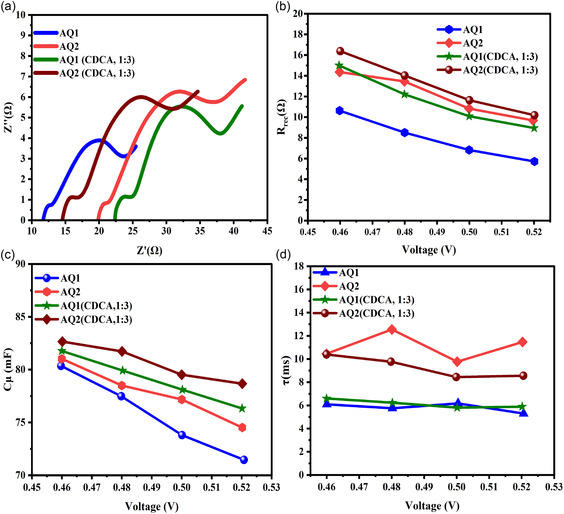
(a) Nyquist plots of AQ1‐ and AQ2‐based DSSCs with and without CDCA (1:3), measured under AM 1.5G illumination. (b) *R*_r__ec_ extracted from EIS, showing higher values for AQ2 and further enhancement with CDCA. (c) *C* *_μ_* versus bias voltage in the dark, indicating CDCA‐induced shifts in charge accumulation. (d) ***τ*** as a function of bias, confirming reduced recombination and longer lifetimes for AQ2, especially with CDCA.

In the absence of CDCA, *R*_rec_ increases from 6.83 Ω for AQ1 to 10.83 Ω for AQ2, demonstrating that the bulky branched alkyl substituents in AQ2 provide effective steric shielding at the TiO_2_ surface (Figure 7a–d). This steric barrier limits electrolyte penetration and reduces the density of surface recombination pathways, leading to improved interfacial charge separation. Upon CDCA coadsorption, both dyes exhibit increased *R*_rec_ values due to enhanced surface passivation; however, the improvement is more pronounced for AQ2, reaching 11.64 Ω. This behavior indicates a synergistic interplay between molecular steric shielding and coadsorbent‐induced surface regulation, resulting in a more uniform dye monolayer and reduced interfacial defect density (Figure 7b). The chemical capacitance (*C* *_μ_*) decreases moderately upon CDCA addition, from 0.92 to 0.73 mF for AQ2 and from 0.88 to 0.58 mF for AQ1 (Figure 7c). This trend is consistent with a slight downward shift of the TiO_2_ conduction band edge induced by interfacial dipole formation, which influences charge accumulation without compromising electron injection efficiency. Importantly, the reduced *C* *_μ_* does not adversely affect device performance, as recombination suppression dominates the overall charge‐transport behavior. Electron lifetime values (*τ* = *R*_rec_ × *C* *_μ_*) further corroborate the superior interfacial properties of AQ2. Under all conditions, AQ2 exhibits longer *τ* values than AQ1, confirming slower recombination kinetics and more efficient charge separation (Figure 7d). Although *τ* decreases slightly upon CDCA incorporation (from 9.96 to 8.58 ms for AQ2), the simultaneous increase in *R*_rec_ and *V*_oc_ indicates that interfacial passivation and energetic modulation, rather than absolute lifetime maximization, govern the overall efficiency enhancement. This observation highlights that optimized interfacial energetics can outweigh modest reductions in *τ* when recombination pathways are effectively suppressed. Although the EIS analysis suggests a slight downward shift of the TiO_2_ conduction band for AQ2, which would typically be expected to reduce *V*_oc_, the experimental devices exhibit an increased *V*_oc_. This apparent contradiction can be rationalized by considering the significantly higher recombination resistance and prolonged electron lifetime observed for AQ2. The bulky branched alkyl substituents provide effective steric shielding at the TiO_2_/electrolyte interface, suppressing back‐electron transfer from the TiO_2_ conduction band to the redox electrolyte. As a result, the steady‐state electron density in the TiO_2_ conduction band increases under illumination, shifting the electron quasi‐Fermi level upward. Consequently, recombination suppression dominates over the minor conduction‐band shift, leading to a net enhancement in *V*_oc_. This behavior highlights the critical role of steric interface engineering in controlling recombination kinetics and optimizing photovoltage in DSSCs.

## 3 Conclusion

This study demonstrates that steric alkyl‐chain engineering can serve as a primary interfacial design strategy, rather than a passive aggregation–suppression approach, to regulate *R*_rec_, *τ*, and photovoltaic efficiency in DSSCs. By comparing two π‐extended unsymmetrical squaraine dyes (AQ1 and AQ2) with similar electronic structures, branched alkyl substituents in AQ2 were shown to provide effective interfacial steric shielding, suppress back‐electron transfer, and enhance *V*_oc_ and *η* without increasing dye loading. Supported by EIS and DFT analyses, these findings establish a mechanistic framework for steric‐controlled interfacial engineering and offer general design guidelines for high‐performance organic sensitizers.

## 4 Experimental Section

All synthetic operations were performed using analytical‐ and spectroscopic‐grade reagents of high purity (≥99.5%), ensuring optimal reliability and reproducibility of the experimental results. Solvents were thoroughly dried and employed under strictly anhydrous conditions, eliminating the need for further purification. The key starting precursors—compound1 [34], compound 6a‐b [35, 36] 1‐iodopropane, dibutyl squarate, (5‐formylthiophen‐2‐yl)boronic acid, and 2‐cyanoacetic acid—were procured from Aldrich and Alfa Aesar and used as received. FTIR spectra were recorded on a PerkinElmer Spectrum Two (4000–400 cm^−1^). ^1^H/^13^C NMR spectra were measured on a Bruker Avance 400 MHz using CDCl_3_ and DMSO‐d_6_. LC/MS data were obtained with an Agilent 6545 Q‐TOF. UV–Vis absorption was measured on a Shimadzu UV‐2600, and PL spectra were collected using a JASCO FP‐8300. Electrochemical studies (CV and DPV) were performed with an Autolab PGSTAT204 potentiostat. Current–voltage (*I*–*V*) measurements were performed using a PET CT200AAA solar simulator under AM 1.5G illumination (100 mW cm^−2^). IPCE spectra were collected with a Newport Quantum Efficiency System (S3 kit). The simulator was calibrated to 100 mW cm^−2^ with a certified silicon reference cell to ensure measurement consistency. EIS was conducted using a BioLogic SP‐300. Measurements were taken in the dark under different applied voltages in the 0.1 Hz–100 kHz range. Dye desorption experiments for AQ1 and AQ2 were conducted in acidic media to evaluate dye loading and adsorption on TiO_2_ films.

### 4.1 Preparation of Compound (2)

Compound 1 (0.75 g, 3.15 mmol) and 1‐iodopropane (1.07 g, 6.30 mmol) were refluxed in CH_3_CN (15 mL) under N_2_ for 22 h. The solvent was removed to give compound 2 (71% yield). ^1^H NMR (CDCl_3_, 400 MHz): δ 0.88(s, 3H), 0.94(t, 2H), 1.40–1.44 (m, 8H), 4.09(t, 2H), 7.52–7.64(m, 2H), 8.45(d, *J* = 7.58 Hz, 1H). ^13^CNMR: δ 10.06, 26.95, 26.97, 42.65, 54.41, 122.18, 127.78, 129.55, 140.88, 151.63, 195.28.

### 4.2 Preparation of Compound (4)

Indolium salt (2) (1.50 g, 3.68 mmol) and compound 3 (0.83 g, 3.68 mmol) were reacted in *n*‐butanol (15 mL) with Et_3_N (0.74 g, 7.36 mmol). Stirred for 23 h, it was then heated at 70°C for 2.5 h. After workup and chromatography (MeOH/DCM), compound 4 was obtained (71%).^1^H NMR (CDCl_3_, 400 MHz). δ0.95(t, 6H), 1.49(m, 2H), 1.56(m, 4H), 1.72(s, 6H), 3.65(t, 2H), 4.19(t, 2H), 5.63(s, 1H), 6.75(d, *J* = 7.58 Hz, 1H), 7.35(s, 1H), 7.38(d, *J* = 7.58 Hz, 1H). ^13^CNMR: 11.82, 14.13, 19.07, 26.89, 28.02, 47.87, 74.03, 82.20, 110.39, 115.31, 126.05, 130.56, 142.37, 142.79, 164.39, 173.88, 188.51, 188.71, 192.46.

### 4.3 Preparation of Compound (5)

Compound 4 (0.48 g, 2.10 mmol) in acetone (CH_3_COCH_3_, 15 mL) was refluxed with 2 N HCl (3 mL) for 10 h. Solvent removal gave compound 5 in 84% yield. ^1^H NMR (DMSO‐d_6_, 400 MHz). δ 0.94 (t, 3H), 1.55 (m, 2H), 1.72 (s, 6H), 3.64 (t, 2H), 5.63 (s, 1H), 7.26 (d, *J* = 8.64 Hz, 1H), 7.52 (d, *J* = 8.64 Hz, 1H), δ 7.77(s, 1H).^13^C NMR: δ 11.86, 20.30, 27.45, 30.39, 49.47, 92.06, 111.86, 114.83, 126.45, 131.78, 143.99, 144.13, 166.85, 175.44, 193.72.

### 4.4 General Synthetic Procedure for Unsymmetrical Squaraines (6a–b)

Equimolar compounds 5a–b and 4 were dissolved in n‐BuOH/toluene (1:1, 20 mL each) and refluxed under N_2_ for 17 h using a Dean–Stark setup. After cooling, solvents were removed, and column chromatography afforded red crystalline unsymmetrical squaraines 7a–b.

### 4.5 Preparation of Dye (7a)

Compound 6a (1.5 g, 8.56 mmol) and compound 5 (3.22 g, 8.56 mmol) were refluxed for 19 h. Column chromatography (silica, MeOH/DCM 3:97) afforded 7a in 86% yield. ^1^H NMR(CDCl_3_‐400 MHz). δ 0.91 (t, 3H), 1.34 (m, 2H), 1.43 (s, 6H), 1.64 (s, 6H), 3.53 (s, 3H), 4.04 (t, 2H), 5.78 (s, 1H), 6.04 (s, 1H), 6.98(d, *J* = 8.54 Hz, 1H), 7.47(tt, *J* = 8.54 Hz, 2H), 7.57(m, 1H), 7.66(m, 2H), 8.82(d, *J* = 8.54 Hz, 1H). ^13^CNMR: δ 17.51, 20.65, 27.54, 31.45, 44.33, 48.33, 69.25, 86.05, 109.89, 115.85, 118.43, 123.17, 125.64, 126.65, 128.39, 130.65, 139.28, 142.06, 143.62, 168.93, 176.82, 187.33.

### 4.6 Preparation of Dye (7b)

Compound 6b (0.95 g, 1.63 mmol) and compound 5 (0.61 g, 1.63 mmol) were refluxed for 20 h as per the general procedure. Purification on silica gel (DCM) afforded 7b in 71% yield. ^1^H NMR‐400 MHz, CDCl_3_: δ0.95(t, 12H), 1.10–1.41(m, 51H), 1.44(m, 4H), 1.59(m, 2H), 1.78(s, 6H), 1.99(m, 2H), 2.93(t, 2H), 4.01(t, 2H), 5.10(s, 1H), 5.83(s, 1H), 6.87(d, *J* = 8.54 Hz, 1H), 6.94(t, *J* = 8.54 Hz, 1H), 7.31(m, 2H), 7.47(m, 2H), 8.42(d, *J* = 3.97 Hz, 1H). ^13^CNMR: δ10.30, 14.04, 14.31, 22.47, 24.83, 29.70, 29.80, 31.54, 32.04, 35.93, 48.03, 51.08, 73.89, 85.31, 109.79, 115.51, 118.12, 124.42, 125.38, 128.53, 130.39, 137.46, 140.67, 141.44, 142.53, 143.39, 167.46, 176.86, 177.31.

### 4.7 Preparation of Dye (8a)

Compound 7a (0.50 g, 0.94 mmol) was dissolved in toluene/methanol (1:1, 4 mL each) in a sealed microwave vessel. 5‐Formylthiopheneboronic acid (0.22 g, 1.41 mmol), K_2_CO_3_ (0.15 g, 1.11 mmol), and PdCl_2_(dppf) (0.022 g, 0.03 mmol) were added under N_2_, with the mixture purged for 25 min. Column chromatography (silica, CH_3_OH/DCM 0.5:99.5) afforded dye 8a in 75% yield. ^1^HNMR (CDCl_3_‐400 MHz). δ0.94(t, 3H), 1.0–1.44(m, 8H), 1.77(s, 6H), 3.47(s, 3H), 4.01(t, 2H), 5.21(s, 1H), 5.96(s, 1H), 6.97(d, *J* = 8.0 Hz, 1H), 7.21(m, 2H), 7.28(t, *J* = 8.54 Hz, 1H), 7.47(t, *J* = 8.54 Hz, 1H), 7.64(d, *J* = 4.0 Hz, 1H), 7.78(d, *J* = 4.0 Hz, 1H), 7.91(s, 1H), 8.75(d, *J* = 8.0 Hz, 1H), 9.91(s, 1H). ^13^CNMR: δ10.36, 18.96, 20.45, 27.63, 30.51, 31.73, 44.55, 48.45, 69.92, 86.77, 108.76, 118.13, 120.44, 122.92, 123.59, 126.41, 126.74, 128.76, 137.94, 138.61, 141.87, 144.13, 154.54, 18.31, 176.78, 177.60, 182.83.

### 4.8 Preparation of Dye (8b)

Compound 7b (0.50 g, 0.53 mmol) was dissolved in toluene/methanol (1:1, 4 mL each) in a sealed microwave vessel. 5‐Formylthiopheneboronic acid (0.33 g, 2.13 mmol), K_2_CO_3_ (0.15 g, 1.11 mmol) were added under N_2_, degassed for 25 min, then PdCl_2_(dppf) (0.022 g, 0.03 mmol) was introduced. Microwave irradiation for 20 min at 70°C, 60 W, followed by silica gel chromatography (CH_3_OH/DCM 1:99) yielded 8b in 77%. ^1^HNMR (CDCl_3_‐400 MHz). δ0.81−0.88(t, 12H), 1.01–1.15(m, 21H), 1.25(m, 6H), 1.30−1.55(m, 23H), 1.78(s, 6H), 1.97(m, 6H), 3.04(t, 2H), 4.06(t, 2H), 5.25(s, 1H), 5.90(s, 1H), 6.906.98(m, 2H), 7.19–7.25(m, 2H), 7.48(d, *J* = 8.54 Hz, 1H), 7.65–7.70(m, 3H), 7.85(s, 1H), 8.54(d, *J* = 8.0 Hz, 1H), 9.91(s, 1H). ^13^CNMR: δ10.49, 14.22, 22.36, 22.81, 24.79, 29.83, 31.54, 31.94, 31.96, 35.98, 48.43, 50.79, 73.81, 86.28, 108.46, 118.63, 120.42, 123.28, 124.43, 126.70, 127.45, 128.45, 137.54, 137.94, 140.56, 141.39, 141.70, 142.56, 144.12, 154.47, 167.39, 175.22, 176.40, 177.55, 182.48.

### 4.9 Preparation of AQ1 Dye

Compound 8a (0.25 g, 0.26 mmol) in CH_3_CN (15 mL) under N_2_ was refluxed with cyanoacetic acid (0.066 g, 0.78 mmol) and piperidine (0.087 g, 0.47 mmol) for 17 h. The mixture was worked up with DCM, washed with dilute CH_3_COOH and water, dried over Na_2_SO_4_, and purified by silica gel chromatography (CH_3_OH/DCM, 4:96) to afford AQ1 dye in 86% yield. ^1^H NMR(DMSO‐*d*_6_‐400 MHz). δ0.79−0.88(t, 3H), 1.27–1.35(m, 2H), 1.49(s, 6H), 1.78(s, 6H), 3.62(s, 3H), 4.06(t, 2H), 5.21(s, 1H), 5.91(s, 1H), 7.23(d, *J* = 8.0 Hz, 1H), 7.25–7.27(m, 3H), 7.37(t, *J* = 8.0 Hz, 1H), 7.80(d, *J* = 12 Hz, 1H), 7.83(d, *J* = 12 Hz, 1H), 7.98(s, 1H), 8.07(d, *J* = 8.0 Hz, 1H), 8.49(s, 1H), 8.54(d, *J* = 8.0 Hz, 1H). ^13^CNMR: δ10.85, 18.47, 20.15, 27.55, 31.34, 44.28, 48.53, 86.70, 110.90, 117.47, 117.85, 120.59, 123.32, 125.11, 126.67, 127.93, 128.65, 134.39, 139.23, 142.32, 142.70, 144.80, 146.70, 153.67, 164.83, 168.78, 174.24, 176.67, 178.22, 209.53. **(*m/z*): [M+H]** ^**+**^ **cald for C**_**38**_ **H**_**35**_ **N**_**3**_ **O**_**4**_ **S: 629.2348; found: 629.2031.**

### 4.10 Preparation of AQ2 Dye

Compound 8b (0.50 g, 0.52 mmol) and CH_3_CN/ chloroform (8:2, v/v; 20 mL total) were used. Cyanoacetic acid (0.13 g, 1.54 mmol) and piperidine (0.087 g, 0.47 mmol) were successively added and then refluxed for 17 h. They were washed successively with dilute acetic acid (CH_3_COOH, 3 × 15 mL) and water (3 × 40 mL) and then dried over anhydrous Na_2_SO_4_. They were urified by silica gel using a CH_3_OH/DCM (5:95, v/v) eluent system to give AQ2 dye in 81% yield. ^1^HNMR (CDCl_3_‐400 MHz). δ0.82−0.88(m, 12H), 1.0–1.15(m, 22H), 1.30(m, 6H), 1.32–1.50(m, 24H), 1.78(s, 6H), 1.98(m, 6H), 3.04(t, 2H), 4.06(t, 2H), 5.25(s, 1H), 5.90(s, 1H),6.90‐6‐98(dt, *J* = 8.0 Hz, 2H), 7.19(dt, *J* = 8.0 Hz, 2H), 7.48(dd, *J* = 8.54 Hz, 2H), 7.65–7.70(m, 2H), 7.85(s, 1H), 8.54(d, *J* = 8.64 Hz, 1H). ^13^CNMR: δ10.70, 14.44, 14.73, 22.62, 22.66, 27.70, 29.80, 29.82,31.6, 32.02, 32.07, 36.09, 37.39, 48.41, 50.77, 64.76, 73.87, 86.97, 109.03, 114.06, 118.62, 119.99, 123.86, 124.20, 124.92, 126.77, 127.39, 128.48, 134.52, 137.57, 140.99, 141.44, 142.39, 144.57, 146.92, 154.85, 167.62, 175.76, 175.84. **(*m/z*): [M+H]** ^**+**^ **cald for C**_**67**_ **H**_**93**_ **N**_**3**_ **O**_**4**_ **S: 1035.6887; found: 1035.6842.**

### 4.11 Theoretical and Computational Approaches (DFT)

DFT and TD‐DFT calculations were performed using Gaussian 09/GaussView 6.0.16 at the B3LYP/6‐311G(d, p) level, incorporating IEFPCM (DCM) solvation. Frontier molecular orbitals, energy gaps, and absorption spectra were analyzed to assess electronic structure and charge–transfer properties [37, 38, 39, 40].

### 4.12 DSSC Fabrication

DSSCs were fabricated following the procedure described in our previous report [41].

### 4.13 Dye Desorption and Dye‐Loading Quantification

Dye loading on TiO_2_ films was determined by dye desorption experiments. After photovoltaic measurements, the sensitized TiO_2_ electrodes were immersed in 0.1 M NaOH solution in H_2_O/ethanol (1:1 v/v) for 30 min to ensure complete desorption of the adsorbed dye molecules. The resulting solutions were analyzed by UV–Vis spectroscopy, and dye concentrations were calculated using the corresponding molar extinction coefficients. The dye‐loading amount (Γ) was determined from the measured concentration and solution volume.

## Supporting Information

Additional supporting information can be found online in the Supporting Information section.

## Funding

This study was supported by Imam Mohammad Ibn Saud Islamic University (Grant IMSIU‐DDRSP2603).

## Conflicts of Interest

The authors declare no conflicts of interest.

## Supporting information

Supplementary Material

## Data Availability

The data that support the findings of this study are available from the corresponding author upon reasonable request.

## References

[1] A. B. Muñoz‐García , I. Benesperi , G. Boschloo , et al., “Dye‐Sensitized Solar Cells Strike Back,” Chemical Society Reviews 50 (2021): 12450–12550. 34590638 10.1039/d0cs01336f PMC8591630

[2] A. Listorti , B. O’regan , and J. R. Durrant , “Electron Transfer Dynamics in Dye‐Sensitized Solar Cells,” Chemistry of Materials 23 (2011): 3381–3399.

[3] K. Kakiage , Y. Aoyama , T. Yano , K. Oya , J. Fujisawa , and M. Hanaya , “Highly‐Efficient Dye‐Sensitized Solar Cells with Collaborative Sensitization by Silyl‐Anchor and Carboxy‐Anchor Dyes,” Chemical Communications 51 (2015): 15894–15897. 26393334 10.1039/c5cc06759f

[4] A. de Souza Goncalves , “A polymer gel electrolyte composed of a poly (ethylene oxide) copolymer and the influence of its composition on the dynamics and performance of dye,” Journal of Power Sources 195 (2010): 1264–1255, 10.1016/j.jpowsour.2009.09.008.

[5] P. Cui and Z. Ling , “Exploring the Impact of Substituents on the Photophysical Properties of Boron Dipyrromethene Dyes for Enhanced Photovoltaic Performance,” Physica Scripta 100 (2025): 025410.

[6] B. K. Korir , J. K. Kibet , and S. M. Ngari , “A Review on the Current Status of Dye‐sensitized Solar Cells: Toward sustainable Energy,” Energy Science & Engineering 12 (2024): 3188–3226.

[7] Y. Li , Q. Qian , S. Ling , et al., “A Benzothiadiazole‐Containing π‐Conjugated Small Molecule as Promising Element for Nonvolatile Multilevel Resistive Memory Device,” Journal of Solid State Chemistry 294 (2021): 121850.

[8] S. A. Al‐horaibi , A.‐B. Al‐Odayni , M. ALSaeedy , et al., “Enhancing Photovoltaic Efficiency with SQI‐Br and SQI‐I Sensitizers: A Comparative Analysis,” Open Chemistry 21 (2023): 20230168.

[9] H. Zhou , M. Aftabuzzaman , Masud , S. H. Kang , and H. K. Kim , “Key Materials and Fabrication Strategies for High‐Performance Dye‐Sensitized Solar Cells: Comprehensive Comparison and Perspective,” ACS Energy Letters 10 (2025): 881–895.

[10] J.‐M. Ji , H. Zhou , and H. K. Kim , “Rational Design Criteria for D–π–A Structured Organic and Porphyrin Sensitizers for Highly Efficient Dye‐Sensitized Solar Cells,” Journal of Materials Chemistry A 6 (2018): 14518–14545.

[11] Y. Gao , L. Duan , S. Guan , et al., “The Effect of Hydrophobic Alkyl Chain Length on the Mechanical Properties of Latex Particle Hydrogels,” RSC Advances 7 (2017): 44673–44679.

[12] J. Alkabli , Y. Q. Almulaiky , and S. A. Al‐horaibi , “Synthesis of Novel Advanced Squaraine Dyes for Improved Efficiency in Dye‐Sensitized Solar Cells,” Applied Materials Today 42 (2025): 102590.

[13] S. A. Haque , S. Handa , K. Peter , E. Palomares , M. Thelakkat , and J. R. Durrant , “Supermolecular Control of Charge Transfer in Dye‐Sensitized Nanocrystalline TiO_2_ Films: Towards a Quantitative Structure–Function Relationship,” Angewandte Chemie International Edition 44 (2005): 5740–5744. 16059951 10.1002/anie.200500363

[14] T. Gerfin , M. Grätzel , and L. Walder , ”Molecular and Supramolecular Surface Modification of Nanocrystalline TiO 2 Films: Charge‐Separating and Charge‐Injecting Devices,“ Progress in inorganic chemistry (1996): 345–393.

[15] F. Xu , T. T. Testoff , L. Wang , and X. Zhou , “Cause, Regulation and Utilization of Dye Aggregation in Dye‐Sensitized Solar Cells,” Molecules 25 (2020): 4478. 33003462 10.3390/molecules25194478 PMC7582523

[16] Y. Wen , W. Zhang , X. Zhu , J. Zhang , and L. Wang , “Interfacial Properties of High‐Order Aggregation of Organic Dyes: A Combination of Static and Dynamic Properties,” Energy 158 (2018): 537–545.

[17] L. Zhang and J. M. Cole , “Dye Aggregation in Dye‐Sensitized Solar Cells,” Journal of Materials Chemistry A 5 (2017): 19541–19559.

[18] M. Pastore and F. De Angelis , “Aggregation of Organic Dyes on TiO_2_ in Dye‐Sensitized Solar Cells Models: An *Ab Initio* Investigation,” ACS Nano 4 (2010): 556–562. 20020758 10.1021/nn901518s

[19] S. A. Al‐horaibi , S. T. Gaikwad , and A. S. Rajbhoj , “Symmetrical and unsymmetrical squaraine sensitizers for dye‐sensitized solar cells: present day advances and future challenges,” Advanced Materials Proceedings 3 (2018): 274–283.

[20] R. Su , L. Lyu , M. R. Elmorsy , and A. El‐Shafei , “Novel Metal‐Free Organic Dyes Constructed with the D‐D|A‐π‐A Motif: Sensitization and Co‐Sensitization Study,” Solar Energy 194 (2019): 400–414.

[21] C.‐Y. Tseng , F. Taufany , S. Nachimuthu , J.‐C. Jiang , and D.‐J. Liaw , “Design Strategies of Metal Free‐Organic Sensitizers for Dye Sensitized Solar Cells: Role of Donor and Acceptor Monomers,” Organic Electronics 15 (2014): 1205–1214.

[22] G.‐G. Luo , H. Lu , Y.‐H. Wang , J. Dong , Y. Zhao , and R.‐B. Wu , “A D‐π‐A‐π‐A Metal‐Free Organic Dye with Improved Efficiency for the Application of Solar Energy Conversion,” Dyes and Pigments 134 (2016): 498–505.

[23] Y. Wu and W. Zhu , “Organic Sensitizers From D–π–A to D–A–π–A: Effect of the Internal Electron‐Withdrawing Units on Molecular Absorption, Energy Levels and Photovoltaic Performances,” Chemical Society Reviews 42 (2013): 2039–2058. 23192709 10.1039/c2cs35346f

[24] C. Qin , W. Wong , and L. Han , “Squaraine Dyes for Dye‐Sensitized Solar Cells: Recent Advances and Future Challenges,” Chemistry – An Asian Journal 8 (2013): 1706–1719. 23596145 10.1002/asia.201300185

[25] L. Zhang , X. Yang , W. Wang , et al., “13.6% Efficient Organic Dye‐Sensitized Solar Cells by Minimizing Energy Losses of the Excited State,” ACS Energy Letters 4 (2019): 943–951.

[26] N. Vlachopoulos , A. Hagfeldt , I. Benesperi , et al., “New Approaches in Component Design for Dye‐Sensitized Solar Cells,” Sustainable Energy & Fuels 5 (2021): 367–383.

[27] J. Alkabli , “Unsymmetrical Squaraine Dyes with Tailored Substituents for High‐Efficiency Far‐Red DSSCs,” Optical Materials. (2026): 117860.

[28] K. R. Graham , P. Erwin , D. Nordlund , et al., “Re‐evaluating the Role of Sterics and Electronic Coupling in Determining the Open‐Circuit Voltage of Organic Solar Cells,” Advanced Materials 25 (2013): 6076–6082. 23897581 10.1002/adma.201301319

[29] T. W. Holcombe , J. E. Norton , J. Rivnay , et al., “Steric Control of the Donor/Acceptor Interface: Implications in Organic Photovoltaic Charge Generation,” Journal of the American Chemical Society 133 (2011): 12106–12114. 21688785 10.1021/ja203235z

[30] T. Zhou , N. Wang , Y. Gao , and X. Li , “Cation–π Interactions in Polymer Science: From Fundamental Insights to Material Applications,” Polymer Chemistry 16 (2025): 2058–2074.

[31] S. S. R. Kommidi , K. M. Atkinson , and B. D. Smith , “Steric Protection of Near‐Infrared Fluorescent Dyes for Enhanced Bioimaging,” Journal of Materials Chemistry B 12 (2024): 8310–8320. 39101969 10.1039/d4tb01281j PMC11353684

[32] Y. Xu , W. Li , G. Wang , et al., ”Carbon Species Doping Overcomes the Limitation of Graphitic Carbon Nitride Toward Frontier Photocatalysis: Precise Division, Synergistic Strategy and Improvement,“ Advanced Energy Materials (2025): e05992.

[33] S. Pradhan , Y. Kurokawa , S. Shaban , and S. S. Pandey , “Squaric Acid Core Substituted Unsymmetrical Squaraine Dyes for Dye‐Sensitized Solar Cells: Effect of Electron Acceptors on Their Photovoltaic Performance,” Colorants 2 (2023): 654–673.

[34] S. A. Al‐horaibi , A. M. Asiri , R. M. El‐Shishtawy , S. T. Gaikwad , and A. S. Rajbhoj , “Indoline and Benzothiazole‐Based Squaraine Dye‐Sensitized Solar Cells Containing Bis‐Pendent Sulfonate Groups: Synthesis, Characterization and Solar Cell Performance,” Journal of Molecular Structure 1195 (2019): 591–597.

[35] S. A. Al‐horaibi , E. M. Garoon , N. A. Bhise , S. T. Gaikwad , and A. S. Rajbhoj , “The Effect of Bis‐Carboxylic Groups of Squarylium Dyes on the Efficiency of Dye‐Sensitized Solar Cells,” Chemical Papers 74 (2020): 1769–1778.

[36] J. Alkabli , Y. Q. Almulaiky , K. Althumayri , and S. A. Al‐horaibi , “Tailored Unsymmetrical Squaraine Dyes for High‐Efficiency DSSCs: Modulating Aggregation and Charge Injection via Alkyl Chain Engineering,” Journal of Power Sources 656 (2025): 238021.

[37] M. D. Mohammadi , F. Abbas , H. Louis , I. O. Amadu , M. Khalid , and T. E. Gber , “Enhancing Photovoltaic Materials: DFT Insights into Structural Modification of Benzo [1, 2‐b: 4, 5‐b1]dithiophene Unit (BDT)‐Based Molecule,” Computational and Theoretical Chemistry 1231 (2024): 114431.

[38] W. A. Zahid , M. F. Ahmad , W. Akram , et al., “Probing the Effect of Acceptor Moiety Engineering in Carbazole‐Based Hole‐Transporting Materials for Efficient Perovskite Solar Cells,” Advanced Theory and Simulations 6 (2023): 2300495.

[39] K. Youssef , M. Allain , C. Cougnon , et al., “Investigating the Impact of Substitution on the Optoelectronic Properties of Benzothiophenone *S*,*S* ‐Dioxide,” New Journal of Chemistry 49 (2025): 15096–15104.

[40] Z. Ziani , S. Cobo , F. Loiseau , et al., “All Visible Light Photoswitch Based on the Dimethyldihydropyrene Unit Operating in Aqueous Solutions with High Quantum Yields,” JACS Au 3 (2022): 131–142. 36711101 10.1021/jacsau.2c00552 PMC9875246

[41] S. A. Al‐horaibi and A. S. Al Zbedy , “Molecular engineering of unsymmetrical squaraine dyes for enhanced photovoltaic performance in dye‐sensitized solar cells: A comprehensive study on light …,” Journal of Molecular Structure 1351 (2025): 144300.

