# Supplementary material for: Sterically Controlled Interfacial Charge‐Transfer Mechanisms in Unsymmetrical Squaraine Dyes for Suppressed Aggregation and Enhanced Performance in High‐Efficiency Dye‐Sensitized Solar Cells
Source: ChemistryOpen. 2026 Apr 20;15(5):e70199. doi: 10.1002/open.70199 (PMC13096587; doi:10.1002/open.70199)
Supplement: Supplementary file 1 — Supplementary Material [file OPEN-15-e70199-s001.pdf]

Supporting Information

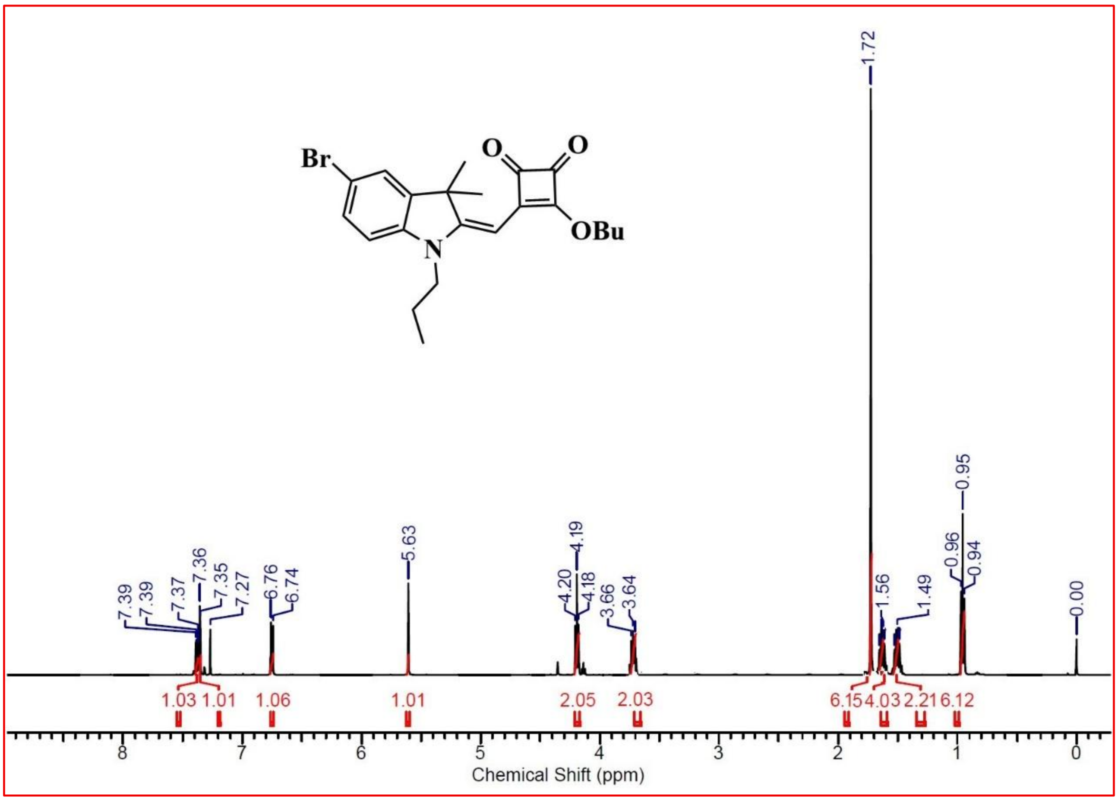

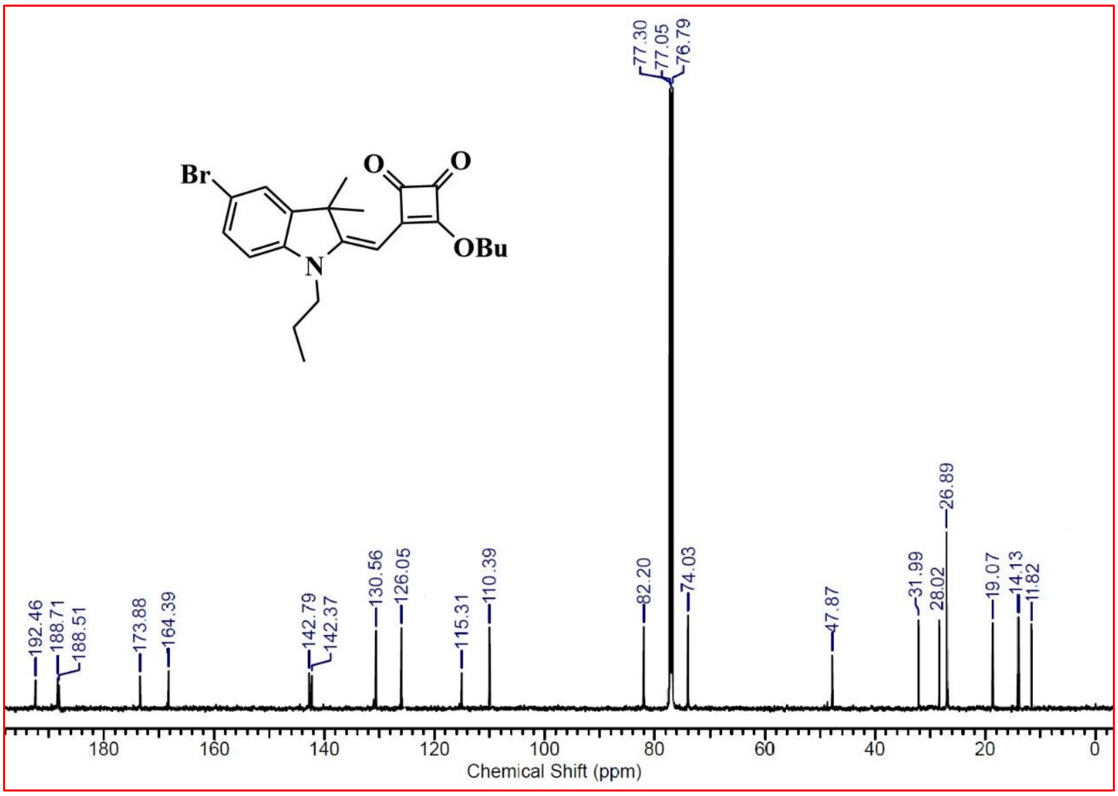

Figure S1: $^{1}H/^{13}C$ NMR of compound (4)

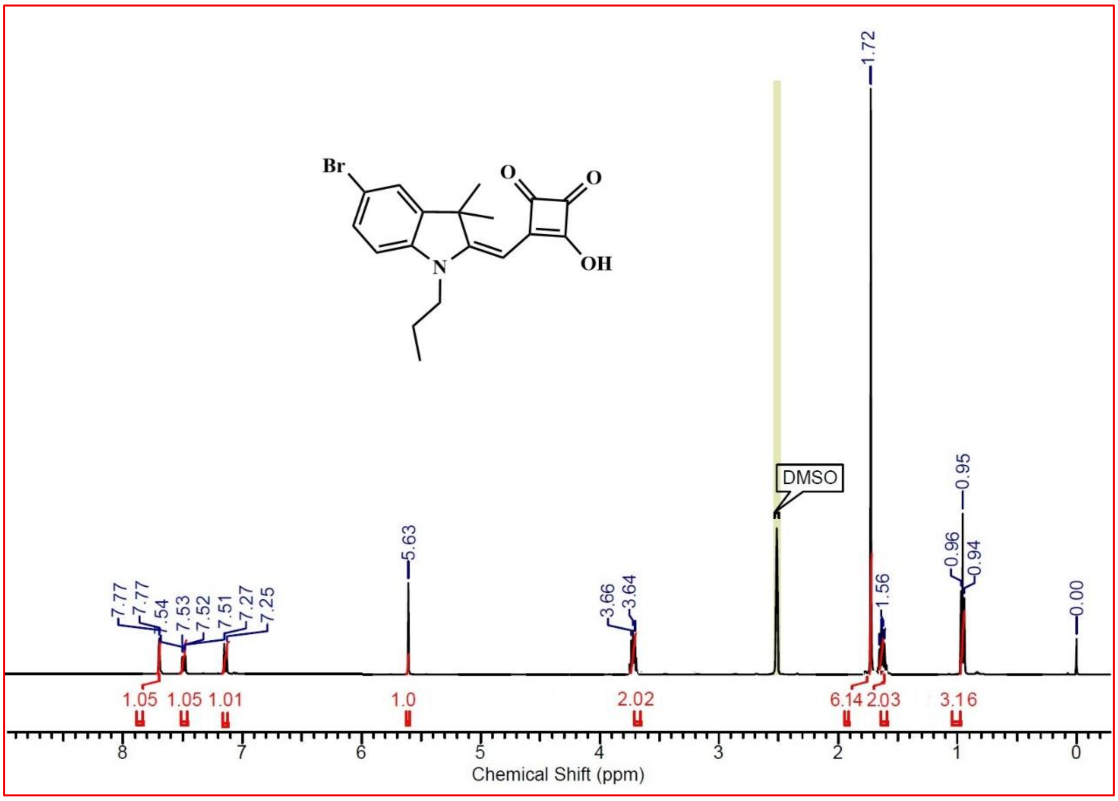

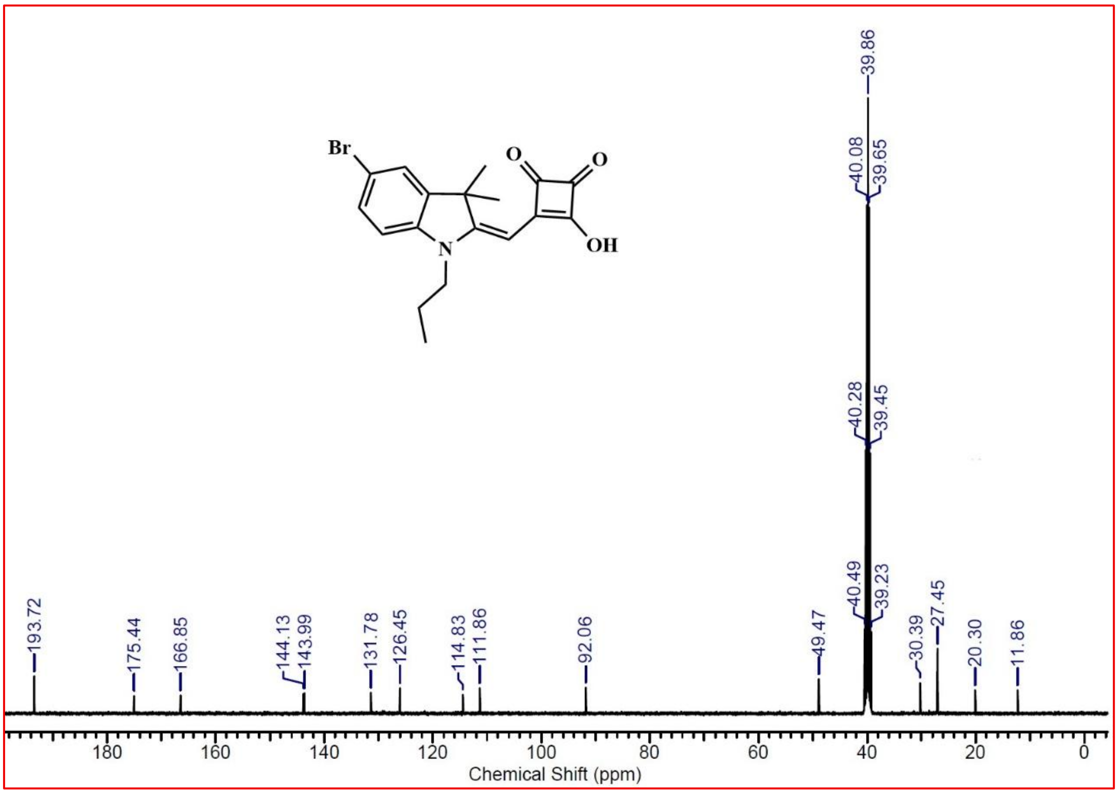

Figure S2: $^{1}H/^{13}C$ NMR of compound (5)

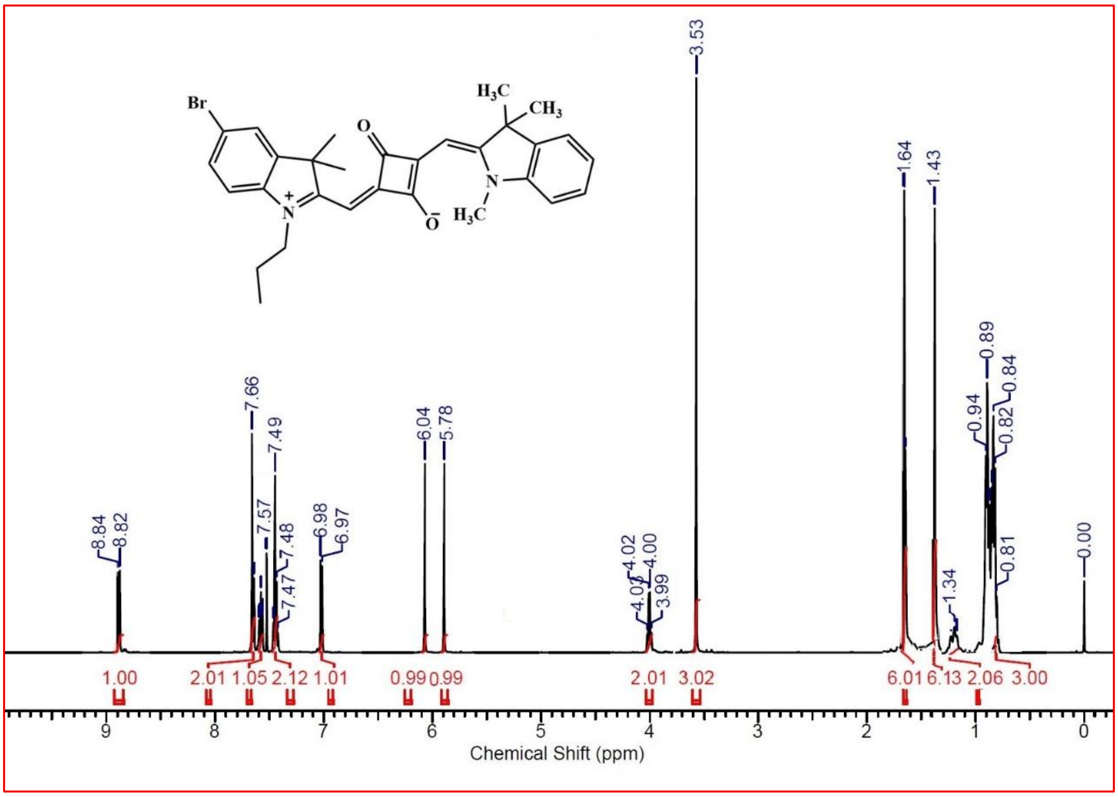

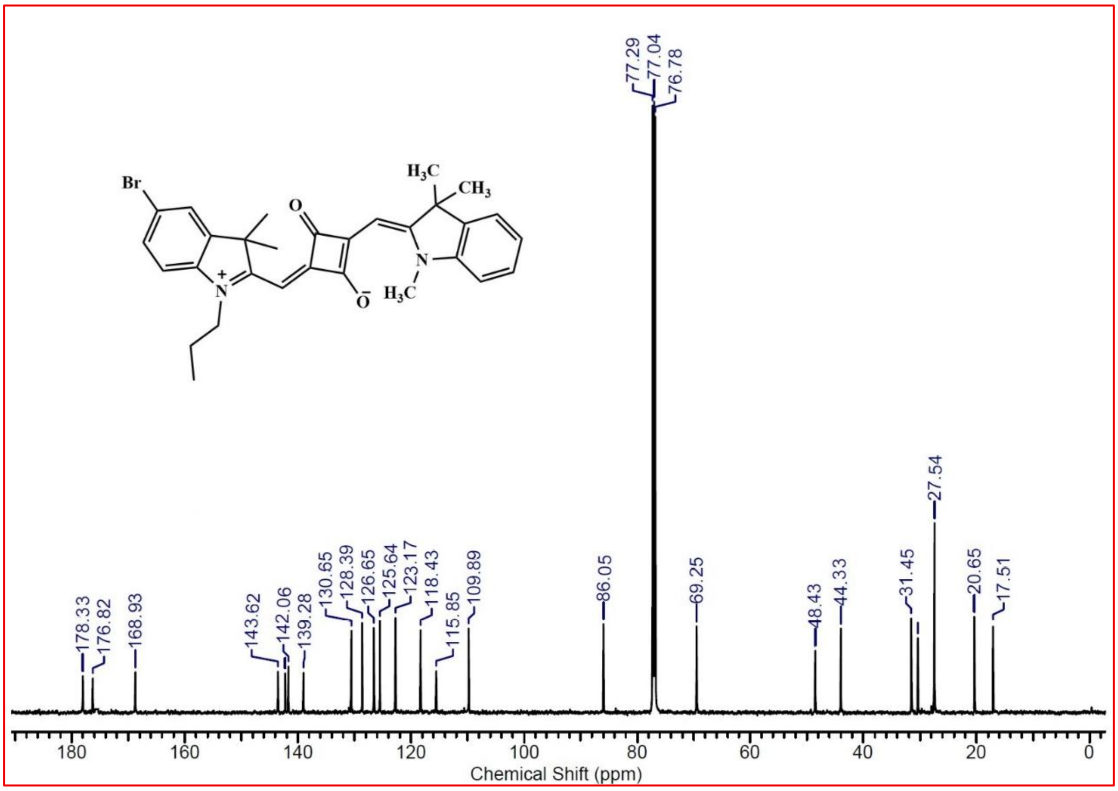

Figure S3: $^{1}H/^{13}C$ NMR of compound (7a)

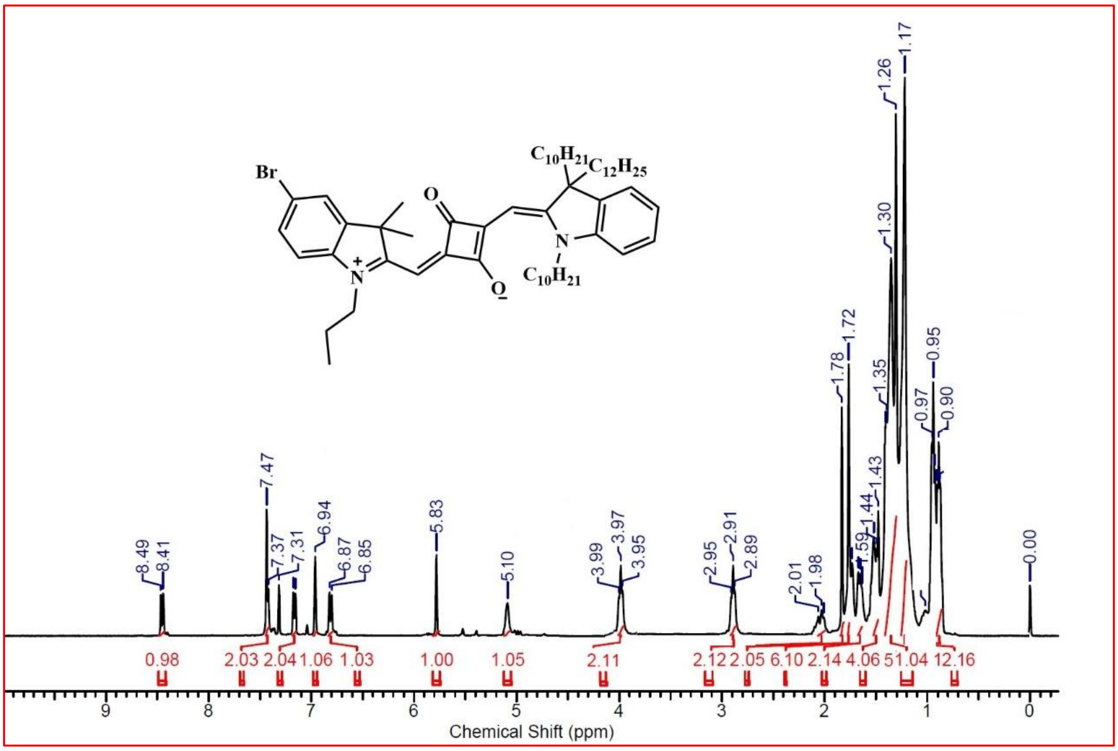

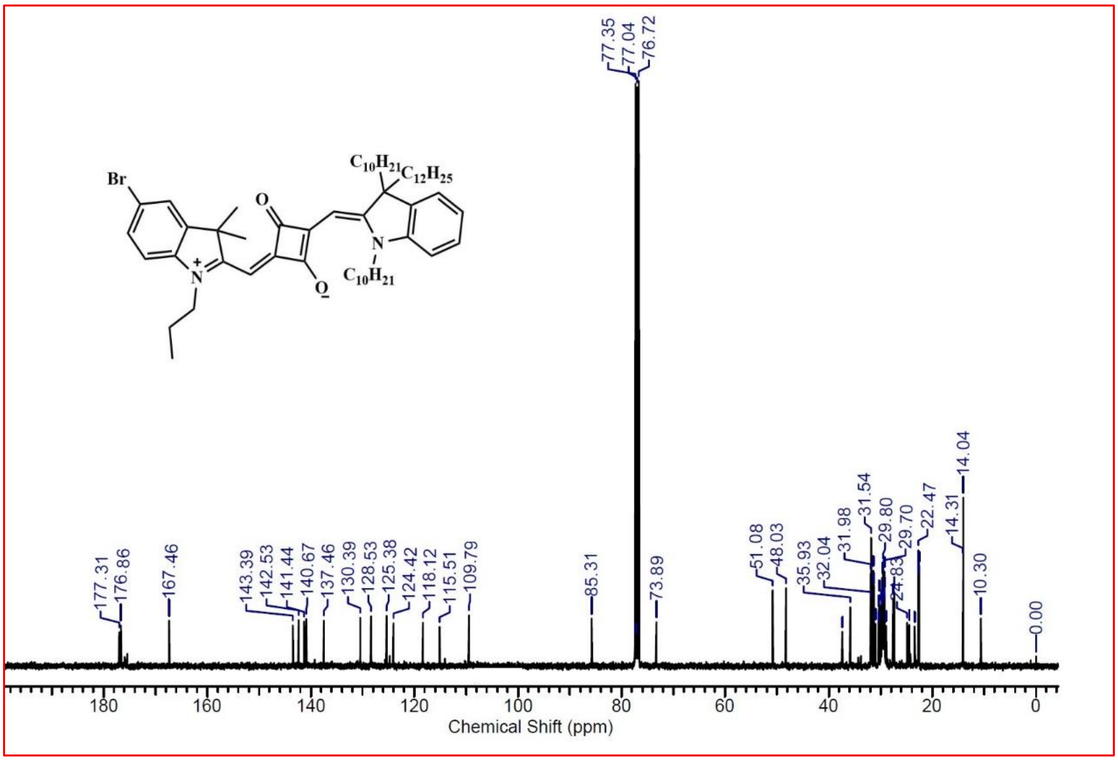

Figure S4: $^{1}H/^{13}C$ NMR of compound (7b)

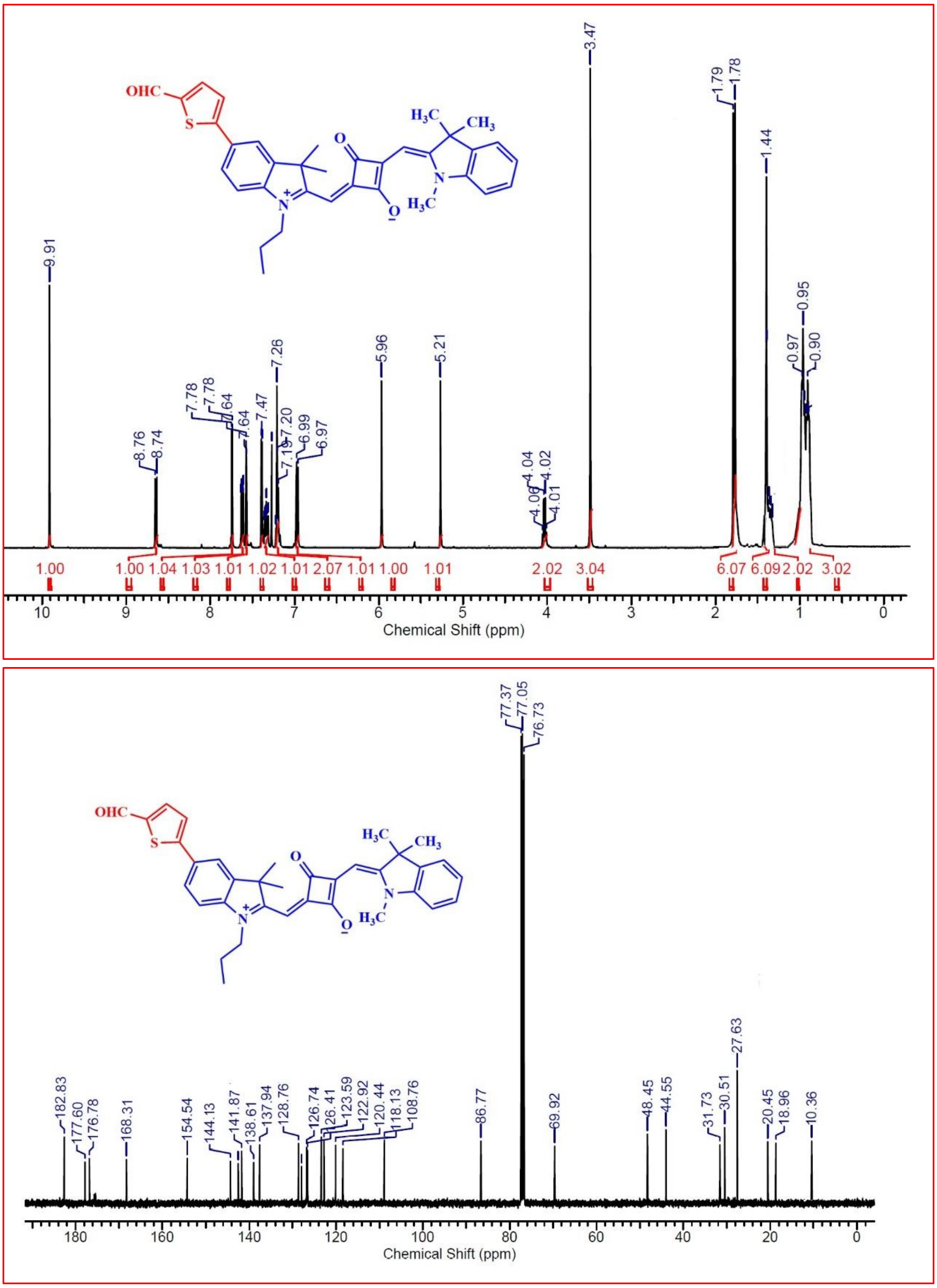

Figure S5: $^{1}H/^{13}C$ NMR of compound (9a)

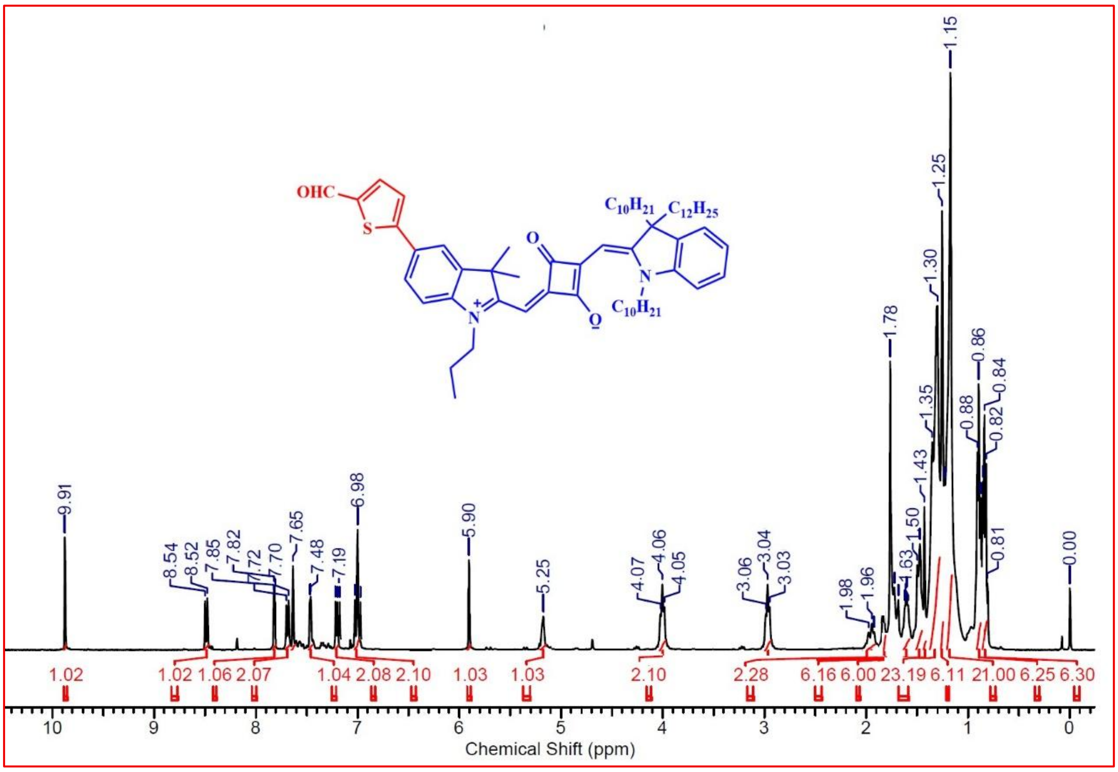

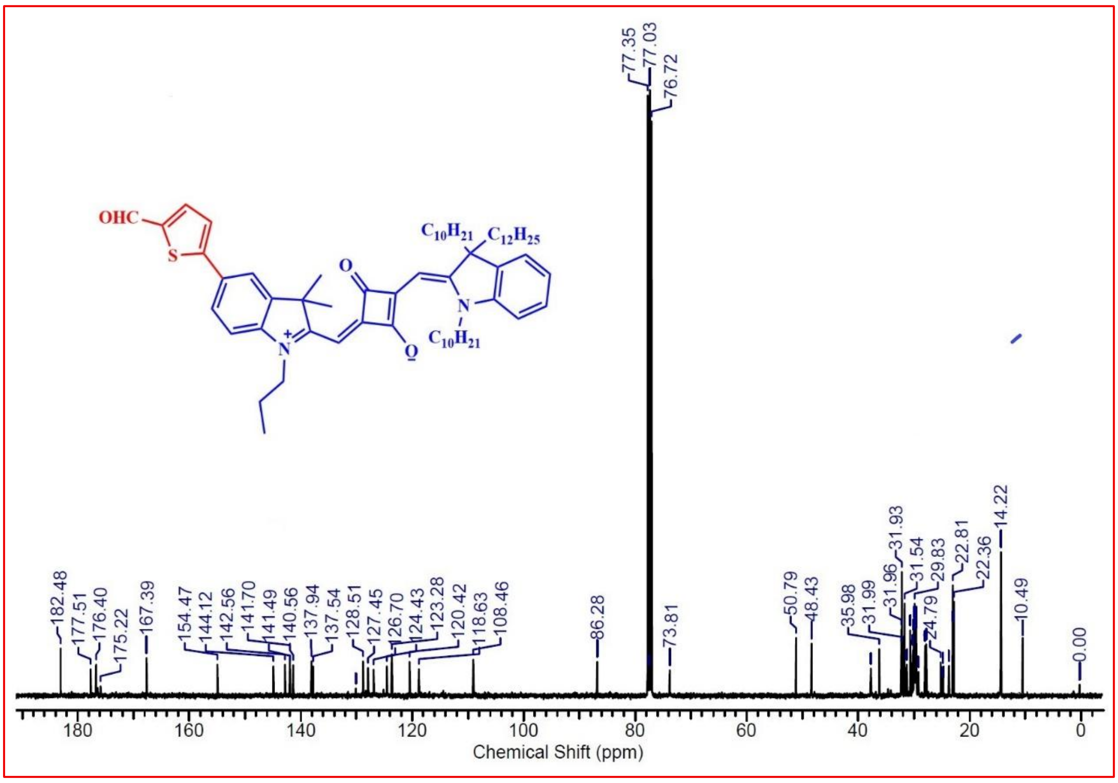

Figure S6: $^{1}H/^{13}C$ NMR of compound (9b)

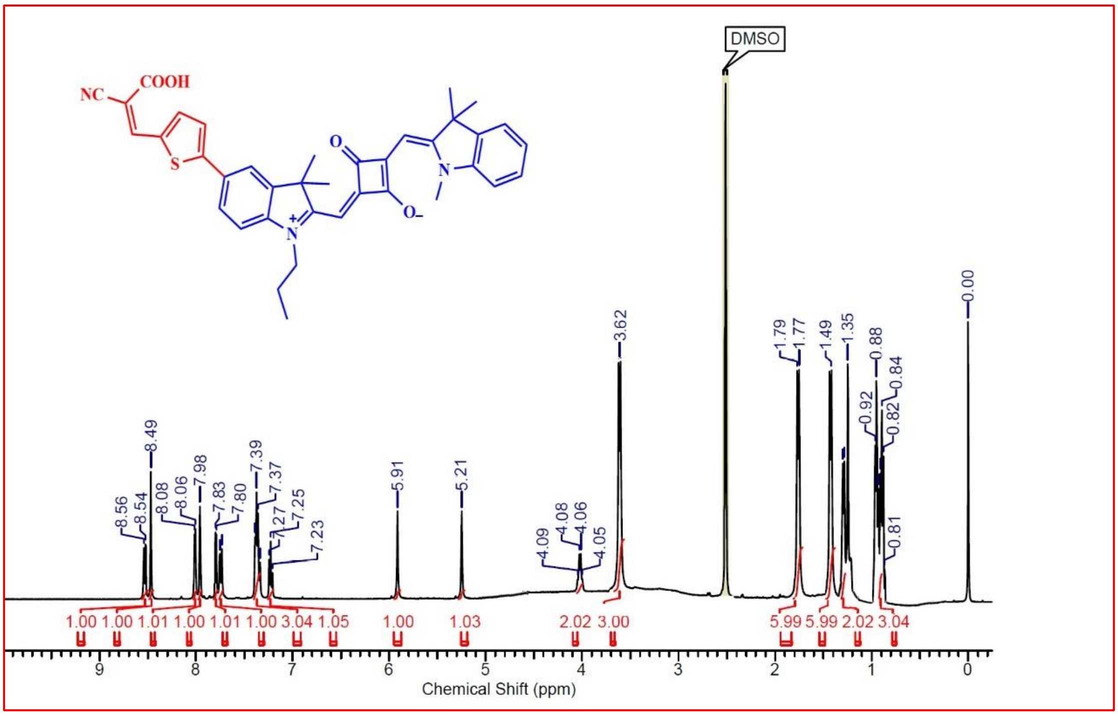

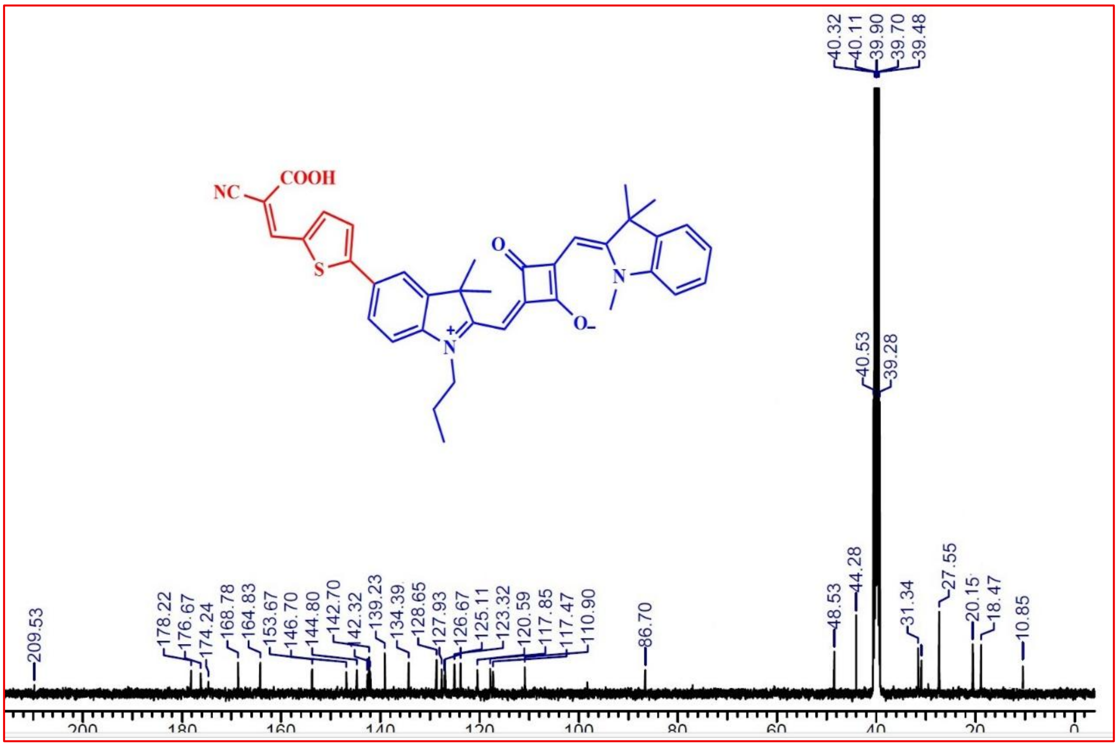

Figure S7: $^{1}H/^{13}C$ NMR of AQ1

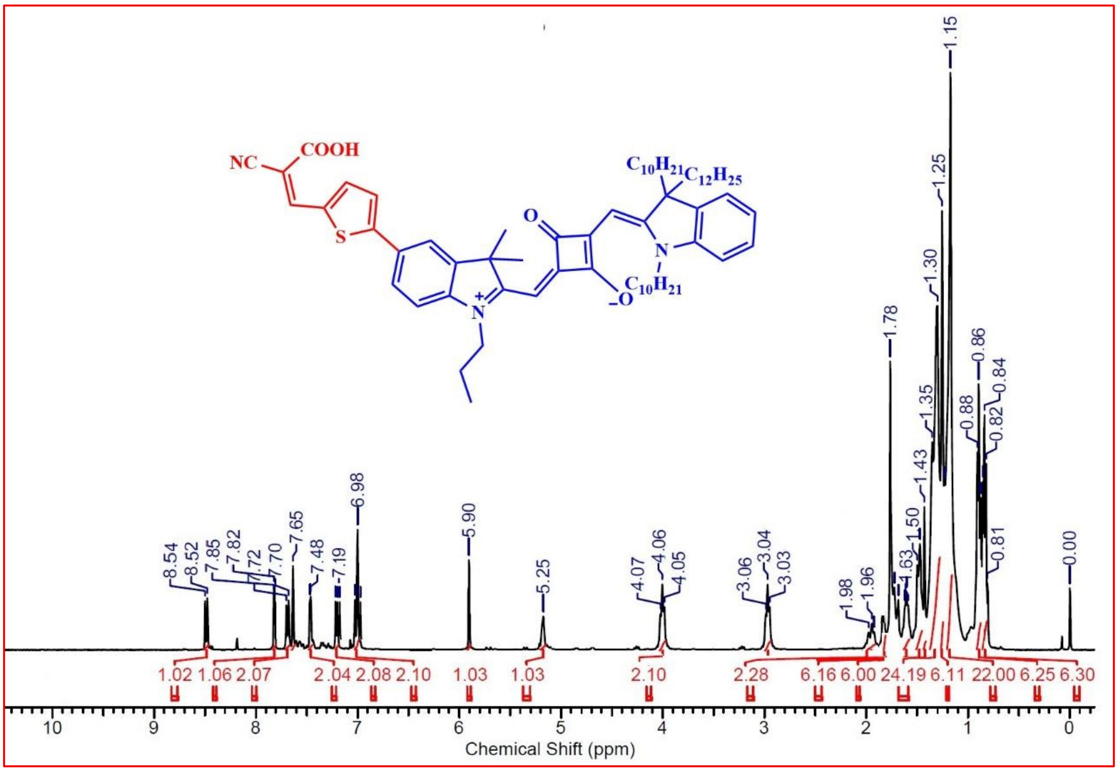

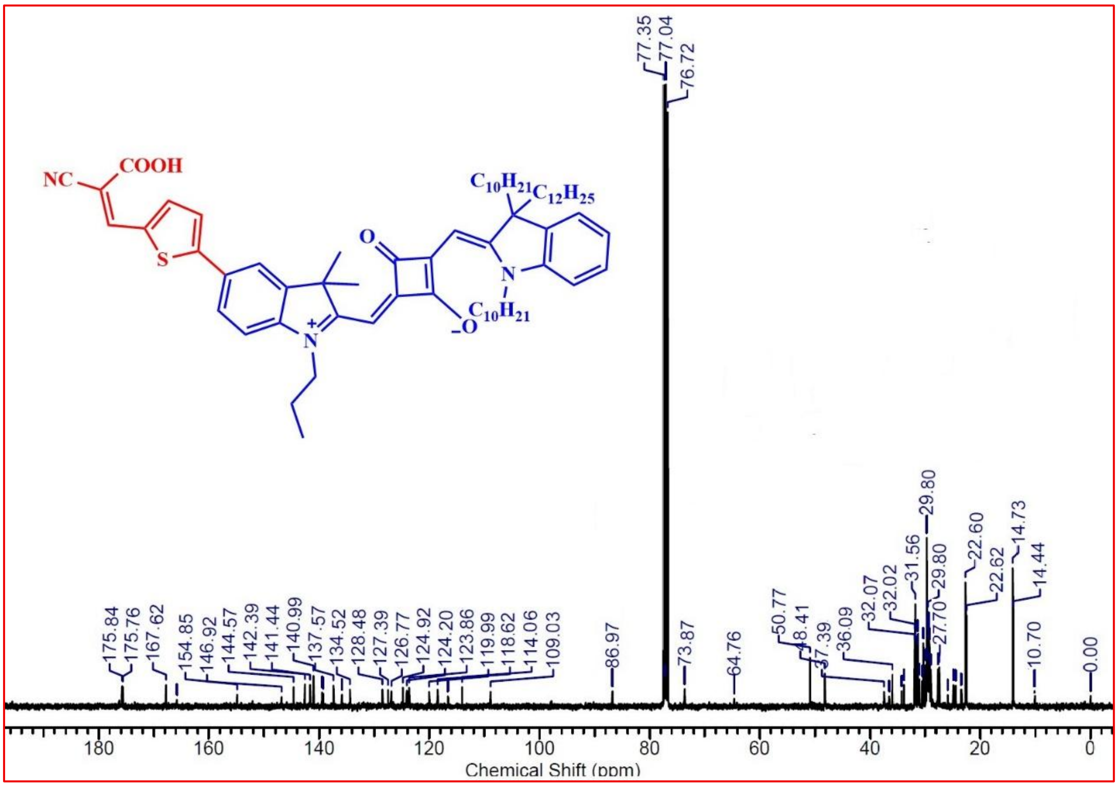

Figure S8: $^{1}H/^{13}C$ NMR of AQ2
